# Viral PIC-pocketing: RSV sequestration of translational preinitiation complexes into bi-phasic biomolecular condensates

**DOI:** 10.1128/jvi.00153-24

**Published:** 2024-02-29

**Authors:** Fatoumatta Jobe, James T. Kelly, Jennifer Simpson, Joanna Wells, Stuart D. Armstrong, Matt Spick, Emily Lacey, Leanne Logan, Nophar Geifman, Philippa Hawes, Dalan Bailey

**Affiliations:** 1 The Pirbright Institute, Woking, Surrey, United Kingdom; 2 Institute of Infection and Global Health, University of Liverpool, Liverpool, United Kingdom; 3 School of Health Sciences, Faculty of Health and Medical Sciences, University of Surrey, Guildford, Surrey, United Kingdom; University of Kentucky College of Medicine, Lexington, Kentucky, USA

**Keywords:** RSV, viral translation, inclusion bodies, RNA viruses, liquid organelles, phase separation, gene expression, pneumoviruses

## Abstract

Orthopneumoviruses characteristically form membrane-less cytoplasmic inclusion bodies (IBs) wherein RNA replication and transcription occur. Here, we report a strategy whereby the orthopneumoviruses sequester various components of the translational preinitiation complex machinery into viral inclusion bodies to facilitate translation of their own mRNAs—PIC-pocketing. Electron microscopy of respiratory syncytial virus (RSV)-infected cells revealed bi-phasic organization of IBs, specifically, spherical “droplets” nested within the larger inclusion. Using correlative light and electron microscopy, combined with fluorescence *in situ* hybridization, we showed that the observed bi-phasic morphology represents functional compartmentalization of the inclusion body and that these domains are synonymous with the previously reported inclusion body-associated granules (IBAGs). Detailed analysis demonstrated that IBAGs concentrate nascent viral mRNA, the viral M2-1 protein as well as components of eukaryotic translation initiation factors (eIF), eIF4F and eIF3, and 40S complexes involved in translation initiation. Interestingly, although ribopuromycylation-based imaging indicates that the majority of viral mRNA translation occurs in the cytoplasm, there was some evidence for intra-IBAG translation, consistent with the likely presence of ribosomes in a subset of IBAGs imaged by electron microscopy. Mass spectrometry analysis of sub-cellular fractions from RSV-infected cells identified significant modification of the cellular translation machinery; however, interestingly, ribopuromycylation assays showed no changes to global levels of translation. The mechanistic basis for this pathway was subsequently determined to involve the viral M2-1 protein interacting with eIF4G, likely to facilitate its transport between the cytoplasm and the separate phases of the viral inclusion body. In summary, our data show that these viral organelles function to spatially regulate early steps in viral translation within a highly selective bi-phasic biomolecular condensate.

**IMPORTANCE:**

Respiratory syncytial viruses (RSVs) of cows and humans are a significant cause of morbidity and mortality in their respective populations. These RNA viruses replicate in the infected cells by compartmentalizing the cell’s cytoplasm into distinct viral microdomains called inclusion bodies (IBs). In this paper, we show that these IBs are further compartmentalized into smaller structures that have significantly different density, as observed by electron microscopy. Within smaller intra-IB structures, we observed ribosomal components and evidence for active translation. These findings highlight that RSV may additionally compartmentalize translation to favor its own replication in the cell. These data contribute to our understanding of how RNA viruses hijack the cell to favor replication of their own genomes and may provide new targets for antiviral therapeutics *in vivo*.

## INTRODUCTION

Orthopneumoviruses, including human and bovine respiratory syncytial virus (RSV) as well as pneumonia virus of mice, are taxonomically classified within the *Pneumoviridae* family and *Mononegavirales* order of negative-strand RNA viruses (1, 2). Human (h) and bovine (b) RSV are globally prevalent and associated with respiratory tract infections, posing significant health and economic burdens (3, 4). Despite their host specificity, the two viruses are genetically and antigenically similar and share orthologous mechanisms of replication and pathogenesis (5–7).

Their small 15 kb non-segmented single-stranded RNA genomes encode 11 proteins from 10 transcriptional units (8), existing as a helical ribonucleoprotein (RNP) complex, together with multiple copies of the nucleoprotein (N) (9). Encoded proteins include those required for autonomous genome replication, mRNA transcription (10), and host immune regulation (11–13). Due to their negative-sense genome, the incoming RNP complex is associated with the RNA-dependent RNA polymerase (L), the phosphoprotein (P), and viral transcription factor (M2-1) (14, 15) required for replication and transcription. Like many negative-sense RNA viruses, genome replication and transcription occur in cytoplasmic membrane-less replication complexes, called inclusion bodies (IBs) (16–18), with the RSV N and P proteins being key drivers of the liquid-liquid phase separation (LLPS) needed for IB formation (16, 19–21) and a potential target for antiviral therapeutics (22, 23).

IBs formed during RSV infection have dynamic properties (23) and host a multitude of viral-viral and viral-host protein interactions underpinning a diverse range of functions (24–27). The best characterized is the concentration of viral proteins for the spatio-temporal compartmentalization of viral RNA replication and mRNA transcription (16–18, 28). Furthermore, a number of host cell proteins involved in the antiviral response [Nuclear factor kappa-light-chain-enhancer of activated B cells (NF-κB) subunit p65 (18), MAVS and MDA5 (29), phosphorylated p38 and O-linked *N*-acetylglucosamine transferase (30)] are sequestered into IBs to prevent their function. Protein phosphatase 1 (PP1) recruitment to the IB has also been shown to be involved in important post-translational regulation steps in the viral life cycle (26).

We and others have also previously shown that IBs are highly organized, with newly synthesized mRNA and the RNA-binding M2-1 protein (18, 31) further compartmentalized into sub-IB droplets, termed inclusion body-associated granules (IBAGs) (17, 18, 31). The presence of poly(A)-binding protein (PABP) and the translation initiation factor eukaryotic translation initiation factor 4 G (eIF4G) in these mRNA-rich droplets (17) suggests a role in translation initiation (32). Although it has been suggested that IBAG contents are released into the cytoplasm for viral translation to occur (17), there is little information available on the detailed steps leading up to the translation of RSV viral mRNAs.

To this end, we used transmission electron microscopy (TEM) and correlative imaging [correlative light electron microscopy (CLEM)] to study RSV modification of the intracellular environment. We observed that IBs induced during infection were structurally heterogenous, with a subset showing bi-phasic organization. Ribosomes were present within microdomains of a limited number of IBs, sites that were subsequently confirmed as synonymous with the previously reported IBAGs. We also observed by immunofluorescence (IF) microscopy that components of the eIF4F and 43S preinitiation complex co-localized with RSV M2-1 and viral mRNA within these IBAGs, likely mediated by eIF4G-M2-1 interactions. Interestingly, mass spectrometry analysis of the spatio-temporal re-organization of host and viral proteins during RSV infection identified significant dysregulation of proteins involved in translation. Furthermore, ribopuromycylation assays combined with immunofluorescence microscopy identified sites of active translation in the IBAGs of infected cells. In summary, our findings represent a novel strategy, preinitiation complex (PIC)-pocketing, by which orthopneumoviruses can hijack components of the cellular cap-dependent translation machinery into viral IBs, data that broadly contribute to our understanding of orthopneumovirus replication.

## RESULTS

### RSV IBs are bi-phasic and spatially re-organize host cell ribosome-like structures into microdomains of reduced electron density

**Fig 1:**
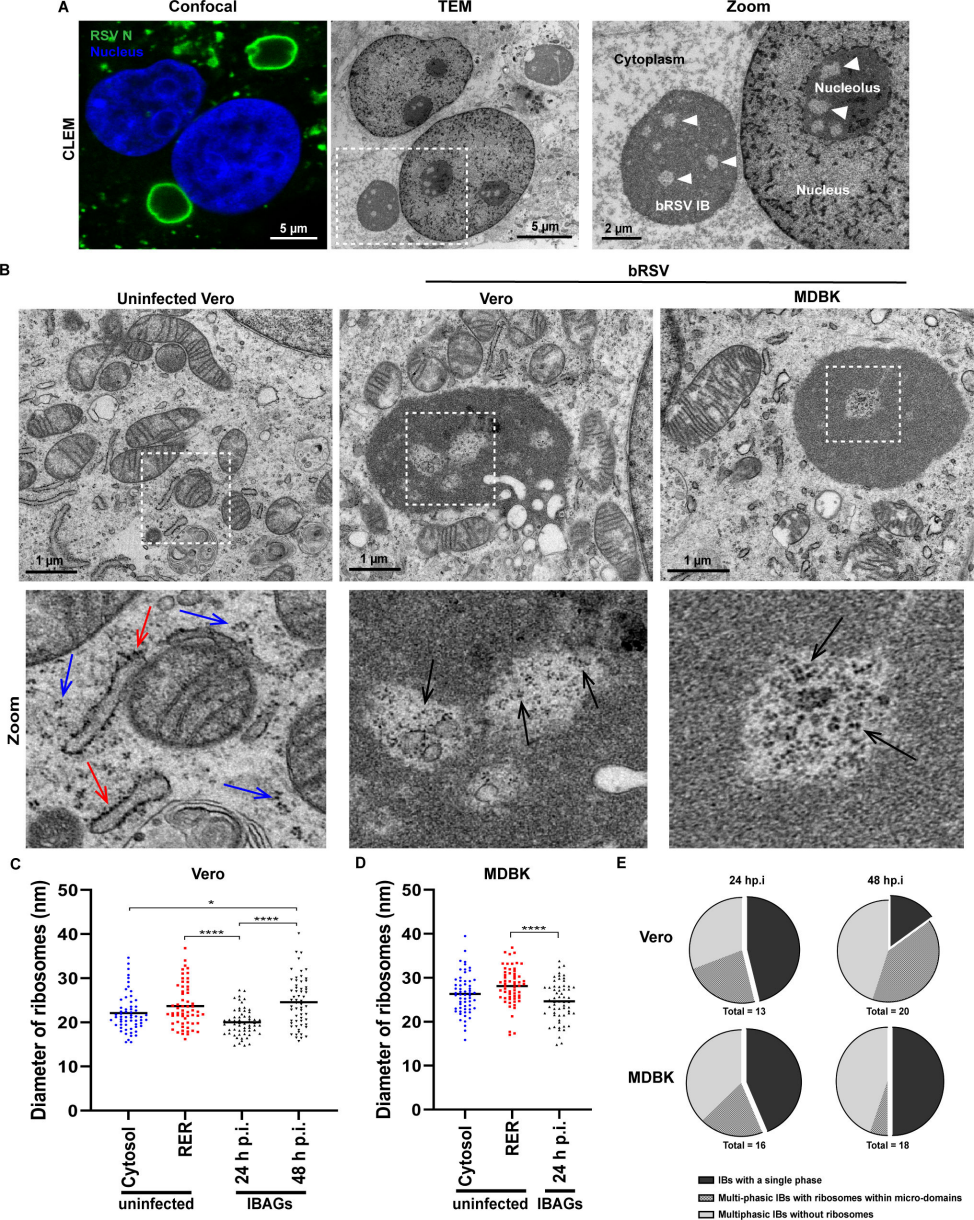
Orthopneumovirus IBs have a bi-phasic morphology, with ribosomes present in microdomains within IBs. (**A**) CLEM. Vero cells infected with bRSV at a multiplicity of infection (MOI) of 1 for 24 h were fixed and immunostained with antibodies against RSV N (green) and nuclei stained with 4′,6-diamidino-2-phenylindole (DAPI) for confocal microscopy. Following confocal imaging, cells were prepared for TEM and the same cells imaged by confocal microscopy (left) identified and visualized by TEM (middle). Zoom (right) shows a higher magnification of the IB and part of the nucleus within the area boxed in the TEM (middle) image. Arrowheads indicate areas of reduced electron density (microdomains) within the IB and nucleolus. (**B**) Uninfected Vero or Vero and MDBK cells infected with bRSV for 24 h were fixed in glutaraldehyde and processed for TEM as detailed in the Materials and Methods. Example images from two independent experiments are shown in the top panel. Zoom (bottom) panel shows higher magnification images of the areas boxed in the top panel. Red arrows indicate ribosomes associated with the rough endoplasmic reticulum (RER); blue arrows, ribosomes in the cytoplasm; and black arrows, clusters of ribosomes in microdomains within IBs. (**C and D**) Comparison of the diameter of ribosomes on the RER and cytoplasm of uninfected cells to ribosomes within microdomains in IBs. Measurements were made of *n* = 60 ribosomes per condition using the Imaris software, as detailed in the Materials and Methods. One-way analysis of variance with Tukey’s multiple comparison was used for statistical analysis; ****P < 0.0001, *P < 0.01. All other comparisons were non-significant. (**E**) Representation of IB heterogeneity. TEM images of IBs in bRSV-infected Vero or MDBK cells at 24 and 48 h post infection (hpi) were assessed for multiphasic organization and the presence of ribosomes within microdomains.

We previously reported that bovine RSV IBs are membrane-less liquid organelles that form by LLPS (18), similar to hRSV IBs (23). Correlative analysis by CLEM again highlighted the ultrastructure of the IBs (Fig. 1A) and the significant modification of the cytoplasmic environment, when compared to uninfected cells (Fig. 1B). Interestingly, however, our analysis of TEM images showed that a subset of bRSV IBs are bi-phasic, with multiple areas of reduced electron density found within the IB itself (Fig. 1A; white arrowheads). We hypothesized that these are synonymous with previously reported IBAG microdomains observed by IF (17, 18). This bi-phasic nature to the IB, to our knowledge, unique in composition and structure within the characterized liquid organelles of non-segmented RNA viruses, shows remarkable similarity with the spatial organization of the nucleolus, a characteristic cellular condensate (Fig. 1A). Of note, microdomains of reduced electron density within the nucleolus (also highlighted with white arrowheads) are fibrillar centers (FCs) where ribosomal RNA transcription occurs (33). It has been suggested that the morphology of bi-phasic condensates is primarily governed by sequence-encoded and composition-dependent protein RNA interactions (34) and likely holds true for our spatially organized RSV IBs. Comparison of bRSV IBs formed in infected Vero (monkey kidney epithelial) or Madin-Darby bovine kidney (MDBK) cells showed similar organization (Fig. 1B and Fig. S1A) validating the use of bRSV-infected Vero cells as suitable models to study orthopneumovirus biology.

Detailed analysis of TEM images of bi-phasic IBs indicated the putative presence of ribosome-like structures in a subset of IBAGs in both bRSV-infected Vero and MDBK cells (black arrowheads in Fig. S1A). Importantly, the putative ribosomes present in IBAGs showed polysome arrangements (black arrows in Fig. 1B, and i in Fig. S1C), similar to ribosomes present within the cytosol of uninfected cells (blue arrows in Fig. 1B and Fig. S1B), providing strong evidence for their identity. In order to provide further confirmation, the longest diameter of ribosomes in the cytosol and on the rough endoplasmic reticulum (RER) of uninfected Vero (red arrows in Fig. 1B) and MDBK cells (red arrows in Fig. S1B) was measured and compared to measurements of the putative ribosomes found within IBAGs following infection. Ribosomes in Vero cells measured a mean diameter of 22.11 nm in the cytosol and 23.70 nm on the RER, compared to 26.32 nm and 28.12 nm in MDBK cells (Fig. 1C and D), highly comparable to the established size range for eukaryotic ribosomes. A similar range of measurements was made in infected cells for each cell type. At 24 h post infection (hpi), “ribosomes” within IBAGs had a slightly smaller mean diameter (20 nm in Vero cells and 24.63 nm in MDBK cells), possibly representing ribosomes in the early stages of assembly (Fig. 1C and D). Structures of a larger diameter were, however, more commonly seen at 48 hpi in Vero cells, more closely aligned with the range observed in uninfected cells. Importantly, ribosomes were not always present in IBAGs, with many having little evidence of these structures (white arrowheads in Fig. S1A, ii in Fig. S1C, and light gray areas in Fig. 1E). Of note, RSV IBs are dynamic liquid organelles that exchange biomolecules with the cytoplasm (17, 23). Thus, IBAG and ribosome composition of IBs is likely highly transient over the course of infection.

**Fig 2:**
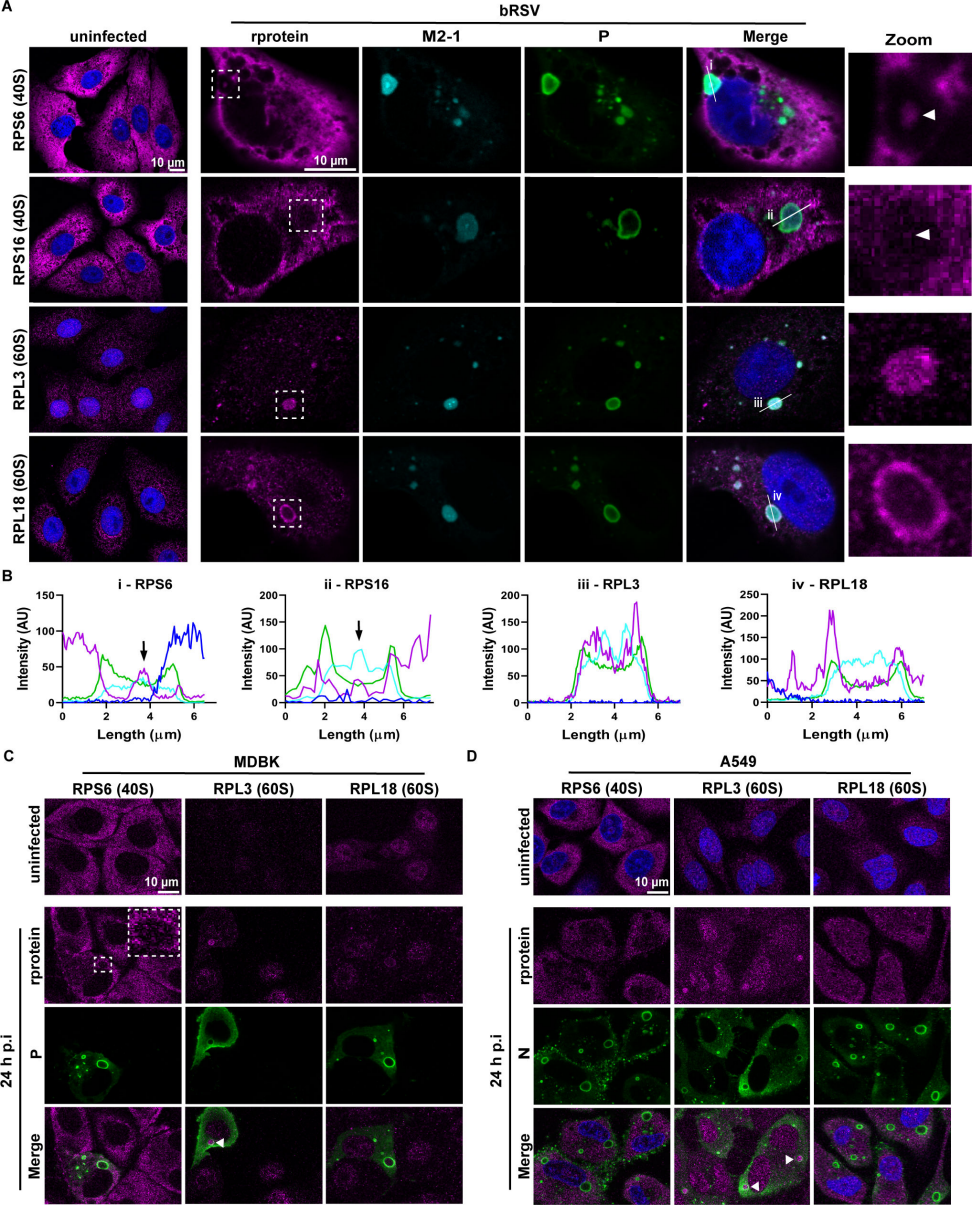
Spatio-temporal characterization of ribosomal subunit proteins by IF microscopy during orthopneumovirus infection. (**A**) Vero cells left uninfected or infected with bRSV at an MOI of 1 for 24 h were fixed, permeabilized, and immunostained with antibodies against ribosomal subunit proteins (rprotein), RPS6, RPS16, RPL3, and RPL18 (magenta), RSV M2-1 protein (cyan), RSV P protein (green), and nuclei stained with DAPI (blue). Images are representative of data from two independent experiments and were obtained using a Leica Stellaris confocal microscope. Zoom panel shows higher magnification of the areas (IBs) boxed in the “rprotein” panel. White arrowheads indicate sub-IB concentration of the rprotein signal, also colocalizing with areas where M2-l protein concentrates. (**B**) Graphs show fluorescent intensity profiles along the white lines drawn in the ‘Merge’ panel in A and labeled i–iv. Black arrows in B indicate co-localization of the rprotein and RSV M2-1. (**C and D**) Uninfected or cells infected with RSV (C, bRSV-infected MDBK cells; D, hRSV-infected A549 cells) at an MOI of 2 for 24 h were fixed, permeabilized, and immunostained with antibodies against RSV N or P protein (green), ribosomal subunits, RPS6, RPL3, and RPL18 (magenta), and nuclei stained with DAPI (blue). Images are representative of data from two (**C**) and three (**D**) independent experiments and were obtained using a Leica Stellaris confocal microscope.

Based on the results of the electron microscopy (EM) analysis, we next explored the spatio-temporal organization of components of the cellular translation machinery during infection using IF microscopy. Protein components of both 40S (RPS6 and RPS16) and 60S (RPL3 and RPL18) ribosomal subunits were examined and found distributed in the cytoplasm of uninfected Vero cells (Fig. 2A). Following infection, both 40S subunits remained mainly located in the cytoplasm; however, we also observed colocalization with RSV M2-1 in IBAGs (white arrowheads in Fig. 2A, and black arrows in i and ii of Fig. 2B). Interestingly, we also observed recruitment of RPL3 into IBs (ring structures of P staining; green) in infected cells (Fig. 2A and iii in Fig. 2B), similar to our previous observations for NF-κB subunit p65 (18). However, recruited RPL3 did not concentrate in IBAGs and RPL18 mainly concentrated at the periphery of IBs (Fig. 2A and iv in Fig. 2B). These data, together with the smaller diameter of ribosomes measured at 24 hpi (Fig. 1C and 1D), suggest that ribosomes in IBAGs may be in the early stages of assembly. However, we also note that the absence of IBAG staining for some subunit proteins may be a result of antibody epitope masking on assembled ribosomes since EM analysis, and the presence of polysomes in IBAGs indicates the presence of translationally active ribosomes. Similar findings were obtained following bRSV infection of MDBK cells (Fig. 2C). Interestingly, despite the use of orthologous replication strategies, we observed little evidence of RPS6 and RPL18 within hRSV IBs unlike RPL3 which was recruited (Fig. 2D)—similar to our observations in bRSV-infected Vero and MDBK cells. Again, this may be because of the transient nature of IBAGs, the proportion of hRSV IBAGs containing ribosomes, or potential antibody epitope masking on ribosomes. However, it is worth noting that, in general, bRSV and hRSV IBs are morphologically very similar under microscopy analysis. By 48 hpi, we noted that the distributions of the proteins remained unchanged (Fig. S2A and B); however punctate structures of RPS6 (cyan arrowhead) were sometimes found in close proximity to IBs in hRSV-infected A549 (human lung epithelial) cells (Fig. S2B). Together, the presented data show that RSV IBs are bi-phasic with multiple areas of reduced electron density that may contain ribosomes/ribosomal subunits, nested within the larger electron dense phase.

### Bi-phasic morphology of RSV IBs correlates with functional stratification of the condensate

**Fig 3:**
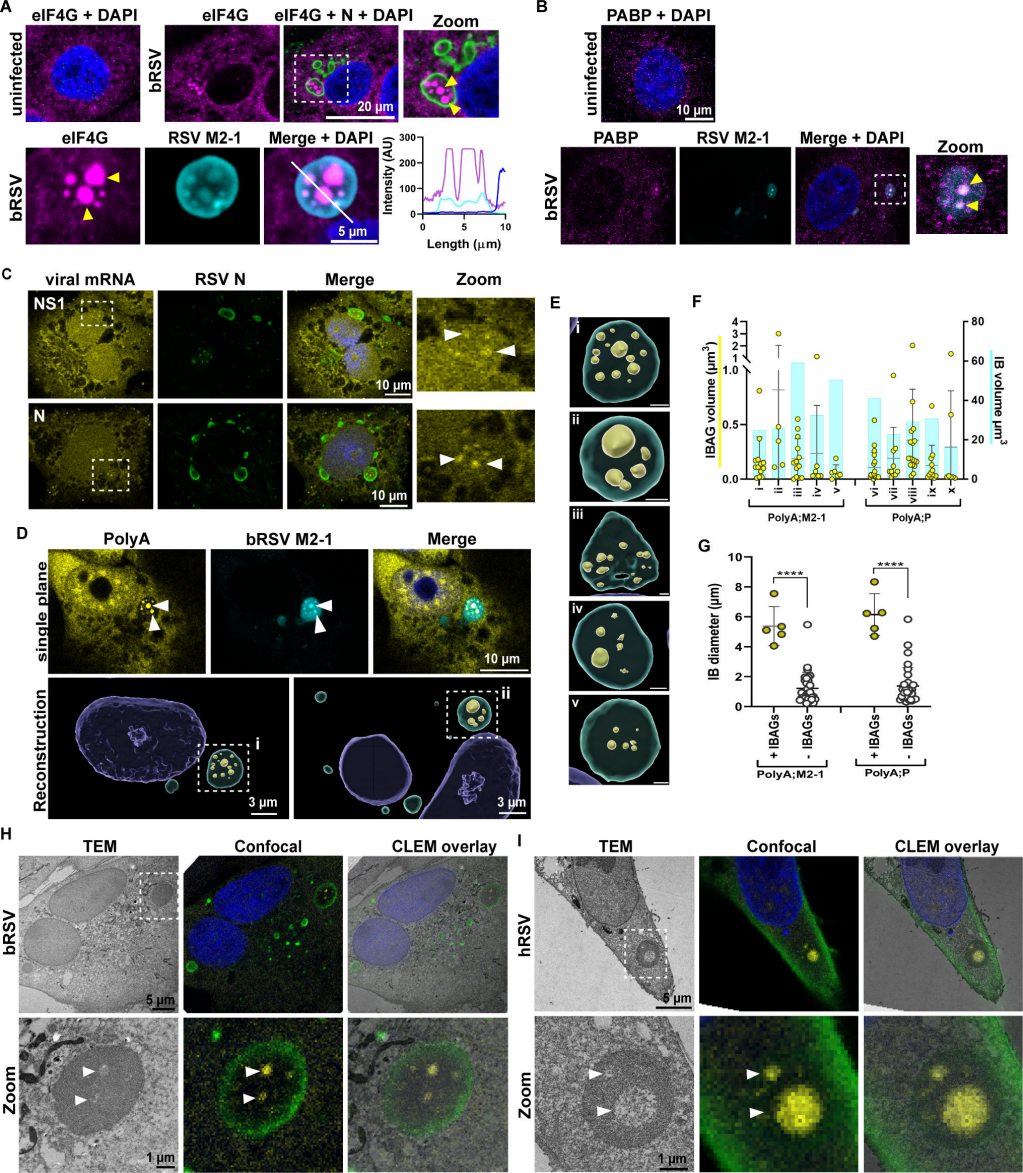
Viral and PolyA mRNA concentrate with eIF4G, PABP, and RSV M2-1 in the differentially phase-separated IB microdomains/IBAGs. (**A and B**) Vero cells were left uninfected or infected with bRSV at an MOI of 1 for 24 h. Cells were then fixed and immunostained for eIF4G (magenta) and RSV N (green) or RSV M2-1 (cyan) in A, and for PABP (magenta) and RSV M2-1 (cyan) in B, and nuclei stained with DAPI (blue) for confocal analysis. Graph shows signal intensities along the white line drawn in the “Merge + DAPI” panel. (**C and D**) Vero cells infected with bRSV at an MOI of 1 were fixed at 24 hpi. FISH analyses were performed (as described in the Materials and Methods) with specific biotinylated probes to detect the indicated viral mRNAs (C; yellow) or total polyadenylated mRNA (D; yellow). Negative controls included uninfected cells stained with the same probes or bRSV-infected cells stained with probes against VSV G mRNA. Co-immunostaining was performed with RSV N (green in C) and M2-1 (cyan in D). Presented images are single-plane confocal images taken in the median section of the cells. Arrowheads indicate sites of mRNA and M2-1 concentration. Bottom panel of D, Imaris reconstruction of confocal image Z-stacks of the same cell from the top panel (left) and another example (right). (**E**) Enlarged images of the IBs boxed in D (**i and ii**) are shown with other examples (iii to v). Scale bars, 1 µm. (**F**) Graph shows volume of the IBs in the panel above (represented as cyan bars) and volumes of the individual IBAGs identified within them (presented as yellow dots), also quantified using the Imaris software, as described in the Materials and Methods. IBs i–v were stained for PolyA and M2-1, and vi–x with PolyA and P. (**G**) Graph shows longest diameter of IBs containing identifiable IBAGs (+ IBAGs) and those without (− IBAGs), also quantified using the Imaris software. An unpaired *t*-test was used for statistical analysis; ****, *P* < 0.0001. (**H and I**) CLEM analysis of (**H**) Vero cells infected with bRSV and (**I**) A549 cells infected with hRSV. TEM was done of the same cells following confocal imaging for PolyA mRNA (yellow) and RSV P (green) and images merged in the CLEM overlay panels. Zoom panels are higher magnification images of the IBs boxed in the panels above.

Building on our observations of ribosomal components within bRSV IBAGs and previous reports of eIF4G, PABP, and total polyadenylated (PolyA) and viral mRNA within hRSV IBs (17), we next examined the locations of all these components in relation to the newly identified bi-phasic nature of IBs (Fig. 1). We first confirmed the recruitment of eIF4G, a key scaffolding component of the eIF4F translation initiation complex, involved in the recruitment of mRNA to the ribosome. In uninfected Vero (Fig. 3A), MDBK (Fig. S3A), and A549 cells (Fig. S3B), eIF4G was distributed throughout the cytoplasm. However, following infection, eIF4G and PABP were recruited into a subset of IBs and spatially concentrated in microdomains (yellow arrowheads) colocalizing with RSV M2-1 in IBAGs (Fig. 3A and B), as previously reported (17). We also previously showed that nascent bRSV viral RNA and bRSV M2-1 protein co-localized in IBAGs during infection (18). Therefore, we sought to confirm the identity of the sub-IB microdomains identified by TEM (Fig. 1) through correlation with IF staining of IBAG markers by CLEM. To identify the best marker of IBAGs for CLEM, we combined fluorescence *in situ* hybridization (FISH) staining with IF microscopy. We observed concentration of bRSV NS1 and N mRNA in small foci (white arrowheads) inside a subset of IBs, in a pattern consistent with IBAGs (Fig. 3C). PolyA FISH and M2-1 co-staining confirmed mRNA accumulation in IBAGs (white arrows in Fig. 3D) and confirmed polyA FISH as the best marker of IBAGs, likely due to its relative higher abundance, when compared to specific viral mRNAs and/or proteins. To develop a deeper understanding of IB morphology, 3D reconstruction analysis was subsequently used to quantify IB and IBAG volumes (see Video S1 and S2). M2-1 (Fig. 3D) or P (Fig. S3C) signals were used as source for IB identification and PolyA for IBAGs (Fig. 3D; insets). These analyses demonstrated the heterogeneity in size and number of IBAGs (Fig. 3E; images i–v and Fig. 3F; also, Fig. S3C). In addition, it provided evidence that IB size is not a determinant of IBAG number and volume. For example, IB i, 24.77 µm³ in volume, has more numerous and larger IBAGs than the larger IB v (50.48 µm³), likely consistent with previous findings that these structures are highly transient in nature (17). Nevertheless, IBAGs were mostly present in IBs with diameter above 4 µm (Fig. 3G). Having established polyA FISH as the best marker for IBAG detection, we used this approach for CLEM analysis of infected cells. Importantly, these experiments confirmed that fluorescent polyA signals (IBAGs) did indeed correlate with sub-IB microdomains of reduced electron density (white arrowheads) in both bRSV-infected Vero (Fig. 3H) and hRSV-infected A549 cells (Fig. 3I), validating our earlier hypothesis and confirming that the bi-phasic nature of IBs relates to RNA- and RNA-binding protein-rich microdomains.

### Components of the translation initiation complex are recruited into the specialized IB microdomains

**Fig 4:**
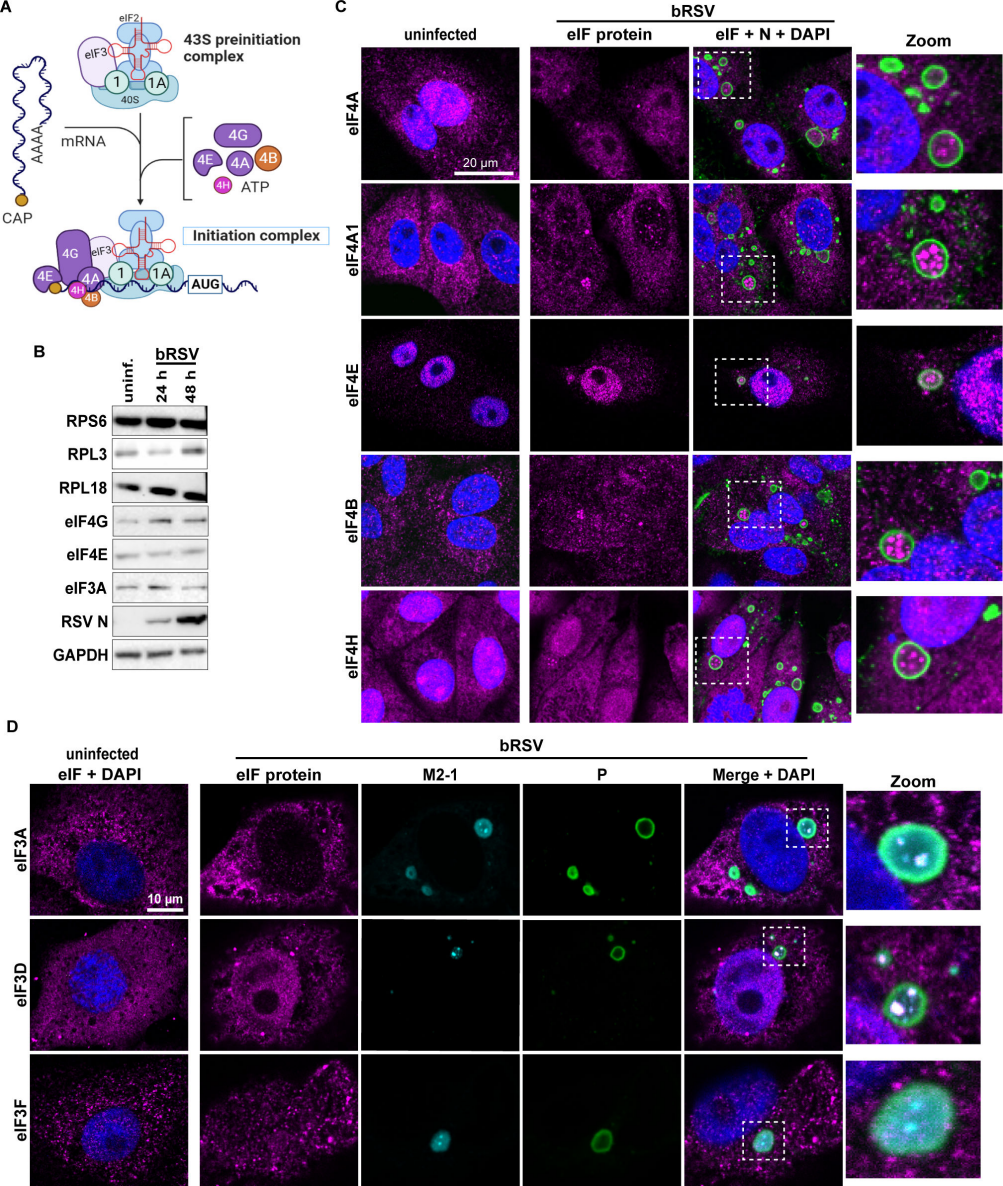
Eukaryotic translation initiation proteins are recruited into microdomains within bi-phasic orthopneumovirus IBs. (**A**) A schematic representation of the association of 43S preinitiation complex with 5´ capped mRNA and components of the eIF4F protein complex to form the initiation complex. Adapted from “Protein Translation Cascade” template in BioRender.com. (**B**) Immunoblots show levels of the indicated proteins in Vero cell lysates—uninfected or infected with bRSV at an MOI of 1 for 24 or 48 h. Blots are representative of two independent experiments. (**C and D**) Vero cells were left uninfected or infected with bRSV at an MOI of 1 for 24 h. Cells were fixed and stained for the indicated eIF proteins (magenta), RSV N in C (green), RSV M2-1 (cyan) and P (green) in D, and nuclei with DAPI in both (blue). Enlarged images of the boxed areas in merged images are shown in the “Zoom” panel. Images are representative of three independent experiments, with the exception of eIF3D and eIF3F, which are taken from a single experiment.

Next, we examined the regulation of other proteins involved in the initiation phase of eukaryotic translation following bRSV infection. Briefly, the eIF4F complex—with eIF4G acting as the scaffold—is essential in the initiation step of protein synthesis, recruiting mRNA to the 43S preinitiation complex through multiple interactions depicted in Fig. 4A, ultimately leading to 80S ribosome formation (35). Whole cell lysate analysis by immunoblotting showed a slight increase in the levels of eIF4G, RPS6, and RPL18 following bRSV infection of Vero cells (Fig. 4B; Fig. S4A). Levels of RPL3 and eIF4E decreased at 24 hpi before increasing at 48 hpi, and no changes were observed for eIF3A. In infected MDBK cells, expression of all assessed proteins were maintained following infection, with the exception of eIF3A decreasing at 48 hpi (Fig. S4B). However, there were notable changes following hRSV infection of A549 and 293T ((human embryonic kidney) cells with levels of all proteins decreasing at 48 hpi due to cell death when cytopathic effects were emerging (Fig. S4B). Nonetheless, these data show that infection does not significantly alter levels of ribosomal and translation initiation complex proteins. By immunofluorescence, in the absence of infection, proteins of the multiprotein complexes eIF4 (Fig. 4C) and eIF3 (Fig. 4D) are distributed in the cytoplasm with varying degrees of nuclear staining. As observed for eIF4G, infection induced their recruitment to sub-IB microdomains, colocalizing with M2-1 in IBAGs as shown in Fig. 4C and D. These data, together with the presence of 40S subunits and viral mRNA, indicate assembly of the initiation complex, depicted in Fig. 4A, within RSV IBAGs.

### IBAGs are functional sites containing ribopuromycylated products

**Fig 5:**
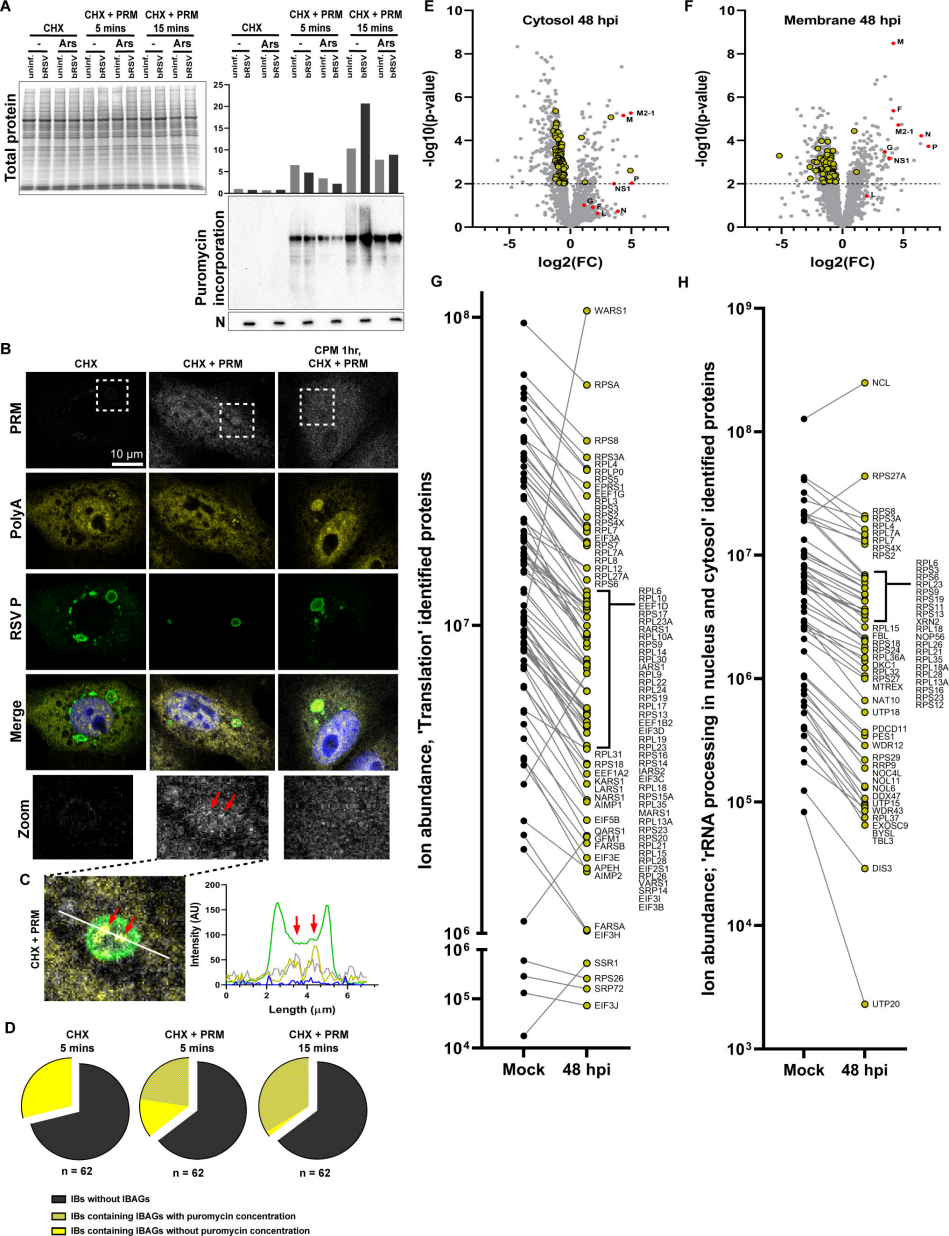
IB microdomains are functional sites containing ribopuromycylated products whose formation correlates with broad dysregulation of the intracellular translation machinery. (**A**) Uninfected or bRSV-infected Vero cells were treated with dimethyl sulfoxide, DMSO (–) or sodium arsenite (Ars) for 1 h, at 24 hpi. Cells were then incubated at 37°C with PRM (to allow incorporation into nascent polypeptides of elongating ribosomes) and CHX (a translation elongation inhibitor) for 5 or 15 min, or with CHX only as control. Whole cell lysates were then prepared to assess total protein levels by Coomassie staining and for levels of ribopuromycylated products by immunoblotting with anti-puromycin monoclonal antibody. RSV N was detected to confirm infection. Graph shows puromycin incorporation (lane signal intensity relative to the uninfected/untreated control sample). The presented data are representative of two independent experiments. (**B**) bRSV-infected Vero cells grown on coverslips were treated with DMSO or CPM for 1 h, followed by CHX only or CHX and PRM for 5 min. Cells were fixed and FISH analysis was performed for PolyA mRNA followed by immunostaining with anti-PRM and RSV P antibodies. A representative image of cells containing IBs with puromycin concentration in IBAGs is presented (CHX + PRM) alongside relevant controls (CHX only and CPM pre-treated). (**C**) Zoom of merged image of IB within boxed area in the middle panel in B (CHX + PRM) along with fluorescent intensity profiles along the drawn white line. Red arrows in B and C indicate areas of PRM and PolyA signal concentration. (**D**) bRSV-infected Vero cells grown on coverslips were treated with CHX, CHX, and PRM for 5 min or 15 min and then fixed with 4% paraformaldehyde for IF analysis. Immunostaining was performed with antibodies against PRM, eIF4G, and RSV P followed by confocal imaging. In each condition, *n* = 62 IBs were imaged and assessed for the presence of IBAGs (using eIF4G staining) and subsequently for the presence of PRM signal within the IBAGs. (**E–G**) Quintuplicate (*n* = 5) membrane (Mem) and cytoplasmic (Cyto) fractions from either mock or hRSV-infected (24 and 48 hpi) cells were isolated using the ThermoFisher Mem-PER Plus Membrane Protein Extraction Kit and analyzed by label-free quantitative mass spectrometry. Protein fractions were run on SDS-PAGE and subjected to in-gel tryptic digestion. Peptides were analyzed on a Thermo Q-Exactive mass spectrometer. Volcano plots reflecting comparison of ion abundancies between mock and infected cytosolic (**E**) membrane (**F**) fractions at 48 hpi are shown with fold change (log2) and *P*-values (−log10 transformed) plotted. The eight successfully detected hRSV proteins are highlighted in red. Proteins with a *P*-value of <0.01 (dotted line at threshold) were subjected to Reactome-based pathway analysis (REACTOME_Pathways_25.05.2022; see Tables 1 and 2). (**E**) The 82 proteins identified from the “Translation” pathway (cytosol fraction; R-HSA:72766; corrected *P*-value 3.40E − 42) and (**F**) the 57 identified proteins from the “rRNA processing in the nucleus and cytosol” pathway (membrane fraction; R-HSA:8868773; corrected *P*-value 9.83E-35) are highlighted in yellow in their respective volcano plots (also see Supplemental Data Set 1). All other proteins are represented by gray symbols. For both pathways, the mean ion abundancies (from *n* = 5 replicates) for each of the associated proteins in both mock and 48 hpi samples are plotted as follows: (**G**) “Translation” pathway proteins and (**H**) “rRNA processing in the nucleus and cytosol” pathway proteins.

RSV infection characteristically induce modification of the cellular microenvironment, with viral RNA replication and mRNA transcription taking place inside IBs. However, there is little evidence available on the spatial organization of RSV mRNA translation. So far, we have shown that viral mRNA, components of the translation initiation complex, and ribosomes may sequester into IBAGs. These observations suggest that translation of viral mRNAs might be occurring in select IBAGs, as well as in the cytoplasm. To assess this, we used ribopuromycylation to interrogate sites of translation during infection. A broader “population level” analysis by Coomassie staining and immunoblotting of whole cell lysates revealed that, unlike many RNA virus infections, global levels of protein synthesis remained unaffected by RSV infection (Fig. 5A; left). It should be noted that since there is no shut-off in host cell translation by RSV, this means that the puromycin (PRM) assay employed here cannot, even indirectly, discriminate translation of host mRNA from viral mRNA. Nevertheless, pre-treatment of cells with the translation inhibitor, NaAsO_2_ (Ars), confirmed the specificity of our labeling by slightly reducing puromycin incorporation translation signals (Fig. 5A; right panel). By IF, co-immunostaining for nascent polypeptide-incorporated PRM, PolyA/eIF4G, and RSV P after 5-min labeling of bRSV-infected cells revealed the majority of the signal was evenly distributed within the cytoplasm (Fig. 5B; Fig. S5A). However, active translation was evident in some IBAGs (red arrows in Fig. 5B and C; Fig. S5A), a signal which was absent when IB organization was disrupted using the condensate hardening agent, cyclopamine (CPM) (Fig. 5B; Fig. S5A). Unsurprisingly, varying PRM signals were observed within IBAGs, from significant to none (Fig. 5D), correlating with our TEM observations on the proportion of IBAGs containing ribosomes (Fig. 1E). Of note, a limitation of the puromycylation assay is the potential release and migration of nascent peptides from ribosomes. This is possibly demonstrated by the higher proportion of puromycin-positive IBAGs following an extended labeling time of 15 min (Fig. 5D) or when cells were pre-treated with puromycin for 30 s before addition of the translation elongation inhibitor, cycloheximide (CHX) (Fig. S5B). Although this approach resulted in more efficient protein labeling (Fig. S5C) compared to concurrent addition of puromycin and cycloheximide (Fig. 5A and B), the latter provided a more accurate representation of active translation sites. Together, these findings demonstrate that IBAGs can act as functional sites for translation, albeit without wholesale effects on translation within the infected cell. Nevertheless, we wanted to understand in more detail how the distribution of cellular translation components changes during infection. To this end, and to examine proteomic changes to the intracellular microenvironment following RSV infection, we performed sub-cellular fractionation of uninfected and hRSV-infected cells at 24 and 48 hpi, using human RSV and human A549 cells because of the better characterized and annotated human proteome. Initial experiments identified satisfactory fractionation of these compartments, validated by Western blot and Coomassie labeling (Fig. S6A and B). Cytosolic and membrane fractions were then harvested from biological replicates (*n* = 5; Fig. S6C) and analyzed by label-free quantitative mass spectrometry (Supplemental Data Set 1A). Principal component analysis (PCA) of the 30 samples showed that component 1 (capturing the largest part of the variation, plotted on the x-axis) showed differentiation between the cytosol and membrane, with this continuing over the duration of the experiment (Fig. S6D). The second component of the PCA captured variation corresponding to time/infection (plotted on the y-axis). In the majority, comparison of the ion abundancies for the successfully detected proteins from the RSV proteome (NS1, N, P, M, G, F, M2-1, and L) demonstrated that RSV predominantly co-fractionates with the membrane fraction from the Mem-PER Kit (Fig. S6E and Supplemental Data Set 1B). Statistical comparison (*t*-test) of the ion abundancies of all detected proteins within the membrane and cytosolic fractions highlighted significant differential regulation of hundreds of cellular proteins at each time point, including well-recognized innate immune restriction factors such as interferon-induced protein with tetratricopeptide repeats 1 (IFIT1, Fig. S6F and Supplemental Data Sets 1C–1J). Subsequent pathway analysis of all significantly, differentially regulated proteins (*P*-value of <0.01) identified numerous enriched pathways, foremost those encompassing ribosome formation, translation, and rRNA processing cellular processes (Tables 1 and 2; Supplemental Data Sets 1C–1J). Interestingly, for the Reactome pathways “Translation” (Table 1 highlighted row; cytosol fraction, R-HSA:72766) and “rRNA processing in the nucleus and cytosol” (Table 2, highlighted row; membrane fraction, R-HSA:8868773) (see Supplemental Data Sets 1K and 1L), we observed that the majority of the identified proteins were downregulated, i.e., present within the negative fold change component of the volcano plot (highlighted in yellow in Fig. 5E and F, respectively). These observations are supported by comparison of the ion abundancies of all the identified proteins in these two pathways in their respective fractions (mock versus 48 hpi; Fig. 5G, “Translation,” *n* = 82; and Fig. 5H, “rRNA processing in the nucleus and cytosol,” *n* = 57). What is surprising about these data is that they point to extensive dysregulation of the host cell translation machinery in infected cells, without obvious wholesale effects on the translational output (see Fig. 5A). To examine this in more detail, we repeated these fractionations and examined a number of proteins well established to play a role in translation by Western blot (Fig. S6G), including three identified in our mass spectrometry pathway analysis as being significantly dysregulated (Fig. S6F; eIF3A, RPL3, and RPS6). In general, we did not observe gross downregulation of these proteins in whole cell lysates, consistent with the lower fold changes observed (Fig. 5G and H). However, we did see significantly modified expression of other components, including eIF4G, eIF3A, RPS6, eIF4B, eIF4H, and RPL3, when comparing the membrane and cytosol fractions (Fig. S6F), highlighting that the intracellular organization of translational components might significantly change during infection, supporting our bioimaging data. Although these experiments were performed in hRSV-infected A549s, our data highlighting the similarity of hRSV and bRSV IBs indicated that similar changes would likely be observed in bRSV-infected bovine cells.

**TABLE 1:** Top 10 Reactome pathways identified for those proteins with a *P*-value <0.01 (Student’s *t*-test) when comparing cytosolic fractions from mock (*n* = 5) with 48 hpi (*n* = 5) samples, ranked by corrected *P*-value^*a*^

Reactome ID	Pathway	Term *P*-value	Corrected *P*-value	No. of proteins identified	% Of pathway identified
R-HSA:72689	Formation of a pool of free 40S subunits	0.00E + 00	0.00E + 00	56	55.45
R-HSA:72706	GTP hydrolysis and joining of the 60S ribosomal subunit	0.00E + 00	0.00E + 00	58	51.8
R-HSA:2408522	Selenoamino acid metabolism	0.00E + 00	0.00E + 00	59	50
R-HSA:156827	L13a-mediated translational silencing of ceruloplasmin expression	0.00E + 00	1.40E − 45	57	51.4
R-HSA:72737	Cap-dependent translation initiation	0.00E + 00	8.41E − 45	58	48.7
R-HSA:72613	Eukaryotic translation initiation	0.00E + 00	8.41E − 45	58	48.7
R-HSA:156842	Eukaryotic translation elongation	0.00E + 00	4.90E − 44	52	55.9
R-HSA:72766	Translation	5.61E − 45	3.40E − 42	82	28.2
R-HSA:9633008	p-T899-EIF2AK4 (GCN2) phosphorylates EIF2AS1	3.50E − 44	2.10E − 41	50	54.3
R-HSA:72672	The 60S subunit joins the translation initiation complex	5.18E − 44	3.09E − 41	49	55.7

^*a*^ The gray highlighted row represents the pathway described in more detail in Fig. 5E and G.

**TABLE 2:** Top 10 Reactome pathways identified for those proteins with a *P*-value <0.01 (Student’s *t*-test) when comparing membrane fractions from mock (*n* = 5) with 48 hpi (*n* = 5) samples, ranked by corrected *P*-value^*a*^

Reactome ID	Pathway	Term *P*-value	Corrected *P*-value	No, of proteins identified	% of pathway identified
R-HSA:8953854	Metabolism of RNA	6.87E − 44	3.68E − 41	108	16.0
R-HSA:8868773	rRNA processing in the nucleus and cytosol	1.83E − 37	9.83E − 35	57	29.4
R-HSA:6791226	Major pathway of rRNA processing in the nucleolus and cytosol	1.35E − 36	7.26E − 34	55	29.9
R-HSA:72312	rRNA processing	3.91E − 36	2.09E − 33	57	27.9
R-HSA:168255	Influenza Infection	2.90E − 29	1.55E − 26	45	28.8
R-HSA:975956	Nonsense-mediated decay (NMD) independent of the exon junction complex (EJC)	2.57E − 28	1.37E − 25	36	37.9
R-HSA:927789	Formation of UPF1:eRF3 complex on mRNA with a premature termination codon and no exon junction complex	2.57E − 28	1.37E − 25	36	37.9
R-HSA:9634669	IMPACT binds GCN1	3.25E − 28	1.73E − 25	35	39.3
R-HSA:9633742	EIF2AK4 (GCN2) dimer autophosphorylates	3.25E − 28	1.73E − 25	35	39.3
R-HSA:9633005	EIF2AK4 (GCN2) binds tRNA	3.25E − 28	1.73E − 25	35	39.3

^*a*^ The gray highlighted row represents the pathway described in more detail in Fig. 5F and H.

### M2-1 plays a role in sub-IB localization of translation initiation factors

**Fig 6:**
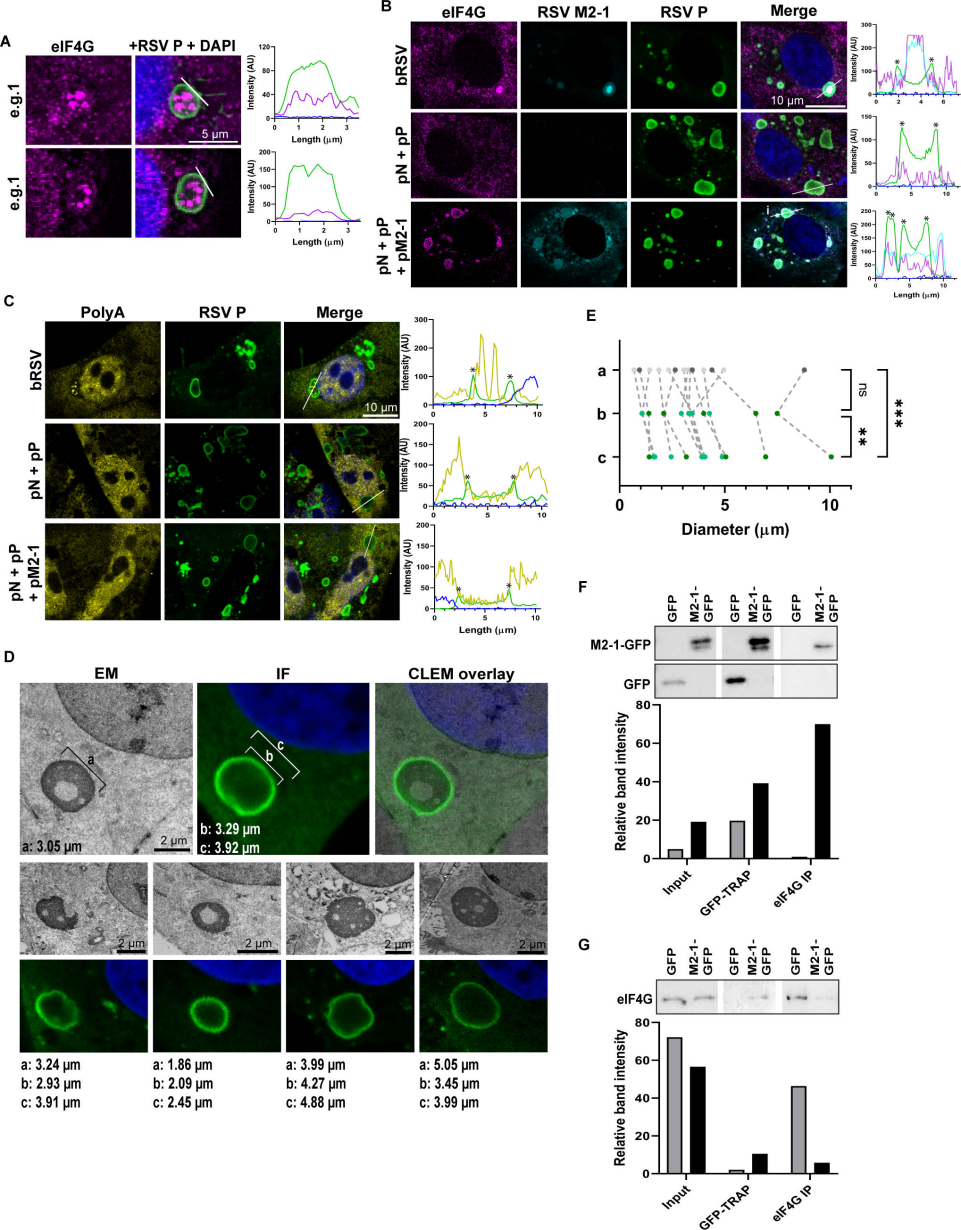
RSV M2-1 protein mediates recruitment of translation initiation proteins into IBs. (**A**) Representative images of bRSV-infected Vero cells immunostained for eIF4G (magenta) and RSV P (green). Graphs show fluorescent intensity profiles along the white lines drawn in the “+RSV P + DAPI” panel. (**B and C**) Vero cells infected with bRSV, transfected with plasmids expressing RSV N and P, or N, P, and M2-1 proteins were fixed and immunostained for eIF4G, RSV M2-1, and P, in B, or FISH labeled for total PolyA mRNA and immunostained for RSV P, in C. Graphs show fluorescent intensity profiles along the white lines drawn in the “Merge” panels. Asterisks indicate areas of increased P intensity at the edge of the IBs. (**D and E**) Using CLEM images of MDBK cells infected with bRSV for 24 h, the longest diameter of IBs was measured in EM micrographs (**a**) and compared to measurements of the internal (**b**) and external (**c**) diameter of the same IBs imaged by confocal microscopy. Panels show examples of five IBs measured. All IBs measured from this experiment are presented in E. One-way analysis of variance with Tukey’s multiple comparison was used for statistical analysis; ***P < 0.001, **P < 0.01, ns, non-significant. (**F and G**) Co-immunoprecipitation. 293T cells were transfected with plasmids expressing M2-1-GFP (green fluorescent protein) or GFP as control. Cell lysates were prepared at 24 h post transfection and incubated with GFP-Trap resin or Dynabeads pre-bound with anti-eIF4G monoclonal antibodies. Bound proteins were analyzed by SDS-PAGE and immunoblotting for the indicated proteins. Data are representative of results from two independent experiments for GFP-TRAP and three for eIF4G immunoprecipitation (IP). The graphs show densitometry analysis of the presented blots relative to background.

Finally, we sought to decipher the mechanism of eIF4F recruitment and spatial organization to these distinct IBAG microdomains inside RSV IBs. We observed that in addition to concentration in IBAGs, eIF4G sometimes accumulated at the IB periphery, where RSV P (Fig. 6A), N, and M2-1 (Fig. 2) can also be found, suggesting an interaction with one or more of these viral proteins. All three viral proteins have been shown to be involved in the recruitment of clients to RSV IBs, e.g., N protein-MDA5 (29), M2-1-PABPC1 (36), and P-RSV M2-1/PP1 (26) interactions. However, the significant co-localization of M2-1 and eIF4F proteins in IBAGs suggested an M2-1-mediated mechanism by which this translation initiation complex is recruited into IBAGs. We have previously identified N and P co-expression as the minimal components for LLPS, with the resultant “pseudo-IBs” recapitulating the p65 recruitment observed during infection (18). Here, we used a similar system to study eIF4G recruitment. We initially found that unlike in infected cells, eIF4G remained at the periphery of pseudo-IBs formed following the co-expression of N and P alone (Fig. 6B). This was consistent and correlated with an absence of IBAGs and the exclusion of polyA signals from pseudo-IBs, presumably since there is no active viral replication in this system (Fig. 6C). However, addition of ectopically expressed M2-1 led to the concentration and co-localization of both M2-1 and eIF4G at the IB periphery, albeit without subsequent IBAG formation (Fig. 6B) or polyA colocalization (Fig. 6C). Together, these findings suggest that spatial re-organization of the IB into bi-phasic functional sites requires components only present during infection; however, M2-1 can sequester eIF4G to the peripheral ring of phase-separated pseudo-inclusions.

**Fig 7:**
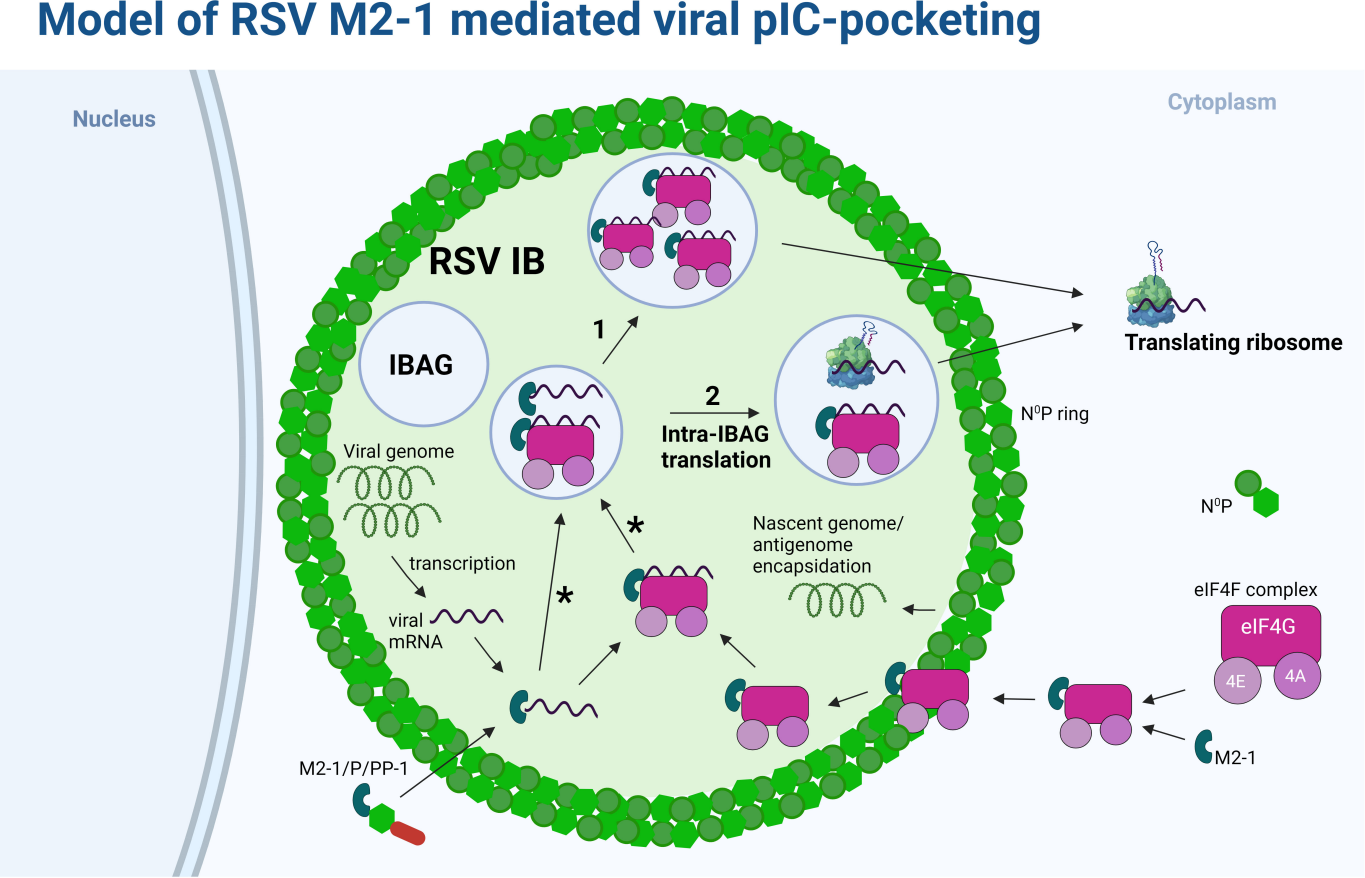
Model of RSV M2-1-mediated viral PIC-pocketing. Cytoplasmic eIF4F complex is recruited to the IB through interactions with the viral M2-1 protein [also recruited through RSV P-PP1 interaction (26)], crossing through a peripheral phase surrounding the IB that is rich in the viral N and P proteins (N^0^P ring). Interactions with nascent intra-IB viral mRNA inside or outside of IBAGs (*) lead to recruitment and enlargement of IBAGs. Translation of the viral mRNAs can take place following complex export into the cytoplasm (route 1) or within the IBAG (route 2). Created with BioRender.com

Parallel live cell analysis of N-GFP and P-derived pseudo-IBs showed they form liquid droplets that undergo fusion (Fig. S7A) and fission events (Fig. S7B) consistent with LLPS and liquid organelle formation. Interestingly, detection of N-GFP and P pseudo-IBs using GFP fluorescence shows even distribution of N throughout the IB, whereas immunostaining with anti-N (in the case of both N and P or N-GFP and P pseudo-IBs) consistently shows a characteristic ring, as observed during IF of infected cells (Fig. S7C). These N ring observations, made by us and others previously, have been attributed to a lack of N antibody staining inside the IB due to poor epitope accessibility (29). Since eIF4G and M2-1 appear to be recruited to this area, perhaps prior to their entrance into the IB, we next turned our efforts to characterizing the nature of this ring in more detail. When we compared measurements of individual IB diameters from our CLEM analysis, we found that the diameter of the phase-separated IBs measured by EM (Fig. 6D; measurement a) was significantly more closely related to the internal (Fig. 6D; b) than external (Fig. 7D; c) diameter of the N ring observed by IF (Fig. 6D and E; Fig. S7D). These data suggest that the ring of N detected by IF is not actually within the phase-separated IB, possibly representing a pool of N protein, perhaps N^0^P at the external periphery of the IB. Of note, although the images used in the analysis are from the same cells, they were taken separately. Nevertheless, efforts were made to acquire both correlated images at the same z-position (in the median section of the cells), using the size of the nucleus as reference.

Finally, to confirm a possible interaction between M2-1 and eIF4G, we performed immunoprecipitation (IP) experiments using cell lysates expressing GFP-tagged M2-1 (M2-1-GFP) or just GFP, as control (Fig. 6F and G). In the first approach using GFP-Trap, eIF4G was found in the bound fraction of M2-1-GFP and not in the GFP only control sample (Fig. 6G) while eIF4G interactors, eIF4E (35) and G3BP1 (37), were not detected in this fraction (Fig. S7E). A parallel IP with eIF4G antibody also revealed M2-1-GFP in the bound fraction and not GFP (Fig. 6F). Attempts to detect eIF4E and G3BP1 in the eIF4G IP pulldowns were unsuccessful due to the presence of IP antibody heavy and light chains in these fractions. However, in summary, these results indicate that a direct or indirect M2-1-eIF4G association underpins the mechanism by which eIF4G and possibly the other eIF proteins are recruited into IBAGs, possibly trafficking through an extra-IB transitionary domain (N^0^P ring) where proteins accumulate before phase separation (Fig. 7).

## DISCUSSION

Orthopneumoviruses, like most negative-sense RNA viruses, are entirely dependent on the host cell translation machinery. Herein, we have demonstrated that bovine and human RSV modulate this machinery, specifically within IBs that exist as bi-phasic condensates, with differential composition and ultrastructure. Furthermore, we have shown that the viral M2-1 protein plays a pivotal role in the sequestration of cellular components to effect this modulation by trafficking cellular proteins from the cytoplasm and between the phases of the viral liquid organelle (Fig. 6).

Initially, the presence of ribosomes and specific ribosomal subunits such as RPS6, RPS16, and RPL3 and viral mRNA within these sites indicated compartmentalization of translation during infection. Later, rearrangement of several proteins involved in translation initiation into IBs and IBAGs together with the presence of ribopuromycylated products clearly demonstrated frequent compartmentalization of translation. Thus, it appears that RSV IBs create cellular microenvironments within which mRNA transcription, viral protein synthesis, and genome replication are segregated, likely advantaging viral replication while protecting viral factors and replication intermediates from host antiviral defenses. Of note, attempts in our laboratory to purify RSV IBs, including the use of differential centrifugation and granule enrichment, IB stabilizing agents such as cyclopamine, (23) as well as biotinylated isoxazole (38), for mass spectrometry-based characterization have so far been unsuccessful. Indeed, it is currently unclear whether either of the fractions in our experimental proteomics analysis (cytosol or membrane) contains any or all of the phase-separated RSV IB.

Similar compartmentalization of the translation machinery has been observed during infection with poxviruses (39), African swine fever virus (40), and reoviruses (41), pointing to conserved viral strategies for subversion of the cell. Interestingly, translational activity is frequently globally suppressed as an antiviral response to infection, via mechanisms such as eIF2α phosphorylation and eIF4G cleavage, which impair translation initiation (42). Indeed, viruses respond by using a diverse range of strategies to selectively subvert cellular translation and/or these antiviral responses toward viral protein synthesis (43–45). Interestingly, however, during RSV infection, we did not observe gross changes to cellular translation kinetics (Fig. 5A). We expected that sequestration of translation initiation components into IBAGs would strongly favor translation of viral protein synthesis over cellular proteins; however, this did not seem to be the case. This may reflect the translational output of the immortalized cell line models used in our experiments and we are currently investigating this recruitment in more physiologically relevant airway models. Nevertheless, our data provide important new evidence that spatially connects translation initiation to the previously reported accumulation of nascent viral mRNA transcripts in IBAGs (17, 18) and also help to shed light on the biophysical implications of this at the ultrastructural level.

Focusing in on these morphologically distinct microdomains [that were confirmed by CLEM to be IBAGs (Fig. 3H and I)], we propose that these areas of reduced electron density form as a result of their distinct molecular and biophysical composition. Although it is currently unclear whether IBAGs are also phase-separated structures driven by the same physiochemical properties as the larger IBs (46), they largely appear spherical in nature (Fig. 1 and 3E; Fig. S1) and were previously shown to be fluid structures (17, 23), two defining features of phase-separated biomolecular condensates (47, 48). Within the cell, it is well established that condensates are frequently formed from networks of RNA and RNA-binding proteins. We propose a distinct composition for the two phases of the bi-phasic IB (see Fig. 7): viral mRNA colocalizing with M2-1 and eIF4F proteins in IBAGs and viral genomic RNA, N, P, M2-1, and L in the rest of the IB, supported by our data on the functional partitioning of the IB. The IBAGs may be less electron dense by TEM because of a higher water content compared to the surrounding IB. This compartmentalization is reminiscent of the spatial organization of the cellular nucleoli into functionally distinct sites, whereby FCs form sites of transcription (33), consistent with our findings for IBAGs. Furthermore, nucleolar spatial organization was shown to represent coexistence of multiple liquid phases, separated by differences in the biophysical properties of their constituent macromolecules (49). The stability of coexisting liquid phases in a ternary complex was shown to be dependent on the presence of a shared component (34). We suggest RSV M2-1 as the candidate partner shared between the two phases of RSV IBs; however, the exact drivers and significance of RSV IB bi-phasic organization require detailed further study.

RSV IBs provide a selective environment where viral processes concentrate. Thus, it is not surprising that their composition would be highly regulated. Condensate components may be segregated into “scaffolds,” proteins that initiate nucleation [RSV N and P proteins in the case of IBs (18)] or “clients,” and proteins subsequently recruited into the structures (46). Nucleation proteins tend to contain specific molecular signatures that support the formation of multiple protein-protein and protein-RNA interactions (48, 50). They may also be involved in the active recruitment of other proteins, for example, M2-1 and PP1 recruitment by the P protein (Fig. 7) (26). Our results showed a previously uncharacterized interaction between eIF4G and M2-1, indicative of a mechanism by which eIF4G and its interacting partners (35) (eIF4E, eIF4A, and eIF3A, etc.; Fig. 4A, C, and D) are recruited from the cytoplasm into IBs, and ultimately IBAGs. Our detailed analysis of CLEM data also identified that a ring of N protein (as well as other scaffolds and clients including eIF4G) may concentrate on the periphery of the IB, and it is attractive to postulate that this is a staging point for proteins about to enter the phase-separated organelle (see Fig. 7). The mechanistic and ultrastructural bases for this LLPS transitionary phase is the subject of further work in our group.

One conflicting aspect of our research remains the determination of the ultimate site for RSV mRNA translation. While we were able to capture and image translation within IBAGs (Fig. 5B), which we think is associated with ribosomal sequestration in IBAGs, our global analysis of translation still points to a majorly cytoplasmic location for this process. It may be that the sensitivity of our assays prevents the correct assessment of translational frequency in IBAGs and that all of these structures contain some ribosomes, allowing a pioneer round of translation to continue, prior to IBAG release from the IB (see Fig. 7; hypotheses 1 and 2). To this end, we are exploring the use of single molecule imaging (at the RNA and protein level) to decipher the exact sites of RSV translation. Future elucidation of the mechanisms underpinning ribosomal recruitment to IBs and IBAG content release into the cytoplasm may also help to improve our understanding of this process.

To conclude, our findings clearly demonstrate compartmentalization of the initiation phase of cap-dependent translation within RSV IBAGs, microdomains which have distinct composition and morphology compared to the rest of the IB. We believe the assembly of eIF4F components and ribosomal subunits on nascent viral mRNA in IBAGs could overcome rate-limiting steps in viral replication, facilitating the outcompeting of cellular transcripts for available translation machinery, somewhat akin to the cap-snatching mechanism of influenza. This process, which we propose be termed “viral PIC-pocketing” is mechanistically underpinned by the viral M2-1 protein which appears a master regulator of the cellular translation machinery.

## MATERIALS AND METHODS

### Cells and viruses

All cells were cultured at 37°C in a 5% CO_2_ atmosphere. A549, MDBK, Vero, 293T, and Hep-2 (human epithelial type 2) cells were obtained from The Pirbright Institute Central Services Unit and maintained in Dulbecco’s modified Eagle’s medium (DMEM; Sigma, Merck) supplemented with 10% heat-inactivated fetal calf serum (FCS; TCS Biologicals), sodium pyruvate (Gibco), and penicillin and streptomycin (Merck). Recombinant bRSV was produced by reverse genetics from bRSV strain A51908 variant Atue51908 (GenBank accession no. AF092942) (51) and hRSV from subtype A (A2 strain). Virus stocks were respectively grown in Vero or Hep-2 cells at 37°C and quantified by 50% tissue culture infectious dose (TCID50) assay.

### Infections and transfections

For virus infections, virus diluted in serum-free medium was adsorbed to cells at MOI of 1 or 2 for 90 min at 37°C in a 5% CO_2_ atmosphere. Uninfected cells were incubated with serum-free medium. Following adsorption, the inoculum was removed, and cells were incubated in medium containing 2% FCS for the indicated times. Plasmids were transfected into cells using 4 µL TransIT-X2 (Geneflow) for every 2 µg DNA according to the manufacturer’s instructions. The plasmids expressing bRSV N (pN) and P (pP) have been previously described (18). Plasmids expressing bRSV M2-1 (pM2-1), M2-1-GFP (pM2-1-GFP), N-GFP (pN-GFP), and GFP alone (pGFP) were constructed by inserting the codon-optimized coding sequences into pcDNA3.1 using the same standard cloning strategy. All sequences were confirmed by conventional Sanger sequencing.

### Antibodies and reagents

Mouse monoclonal antibodies raised against bRSV N (mAb89), P (mAb12), and M2-1 (mAb90) were previously described (52, 53). Rabbit monoclonal, anti-RPL3, and anti-RPL18 were purchased from Affinity Biosciences. Rabbit anti-RPS6 (2217), anti-eIF4G (2467), anti-eIF4E (2067), anti-eIF3A (3411), anti-eIF4A (2013), anti-eIF4A1 (2490), anti-eIF4B (3592), anti-eIF4H (3469), and anti-GAPDH (5174) were obtained from Cell Signalling Technology (CST). Mouse anti-PABP (ab6125) was obtained from Abcam, anti-G3BP-1 was from BD Biosciences, rat anti-GFP was from BioLegend, and Rabbit anti-RPS16 (HPA064222), mouse anti-puromycin, and sodium arsenite were purchased from Sigma, Merck. Secondary horseradish peroxidase-linked antibodies were obtained from CST and Alexa Fluor secondary antibodies were from Life Technologies.

### Quantitative mass spectrometry

In-gel digestion was similar to the described protocol (54). Fractionated samples (normalized to 20 µg) were run approximately 1 cm into a 4%–12% NuPage gel (Invitrogen) before staining with Coomassie blue (GelCode Blue Safe Protein Stain, Fisher) for 2 h, then de-stained with ultrapure water. The entire lane length (1 mm wide) was excised and cut into smaller pieces (approximately 1 mm^3^) before de-staining with 50% acetonitrile (vol/vol) in 25 mM ammonium bicarbonate. Proteins were reduced for 10 min at 60°C with 10 mM dithiothreitol (Sigma, Merck) in 50 mM ammonium bicarbonate and then alkylated with 55 mM iodoacetamide (Sigma, Merck) in 50 mM ammonium bicarbonate at room temperature for 30 min in the dark. Gel pieces were washed for 15 min in 50 mM ammonium bicarbonate and then dehydrated with 100% acetonitrile. Acetonitrile was removed and the gel plugs were rehydrated with 0.01 µg/µL proteomic grade trypsin (Thermo) in 50 mM ammonium bicarbonate. Digestion was performed overnight at 37°C. Peptides were extracted with 50% (vol/vol) acetonitrile, 0.1% trifluroacetic acid (TFA; vol/vol), and the extracts were reduced to dryness using a centrifugal vacuum concentrator (Eppendorf). Peptides were re-suspended in 3% (vol/vol) methanol and 0.1% (vol/vol) TFA for NanoLC mass spectrometry (MS) electrospray ionization (ESI) MS/MS analysis.

LC-MS/MS analysis was similar to that described (55). Peptides were analyzed by on-line nanoflow LC using the Ultimate 3000 nano system (Dionex/Thermo Fisher Scientific). Samples were loaded onto a trap column (Acclaim PepMap 100, 2 cm × 75 µm inner diameter, C18, 3 µm, 100 Å) at 9 µL/min with an aqueous solution containing 0.1% (vol/vol) TFA and 2% (vol/vol) acetonitrile. After 3 min, the trap column was set in-line an analytical column (Easy-Spray PepMap RSLC 50 cm ×75 µm inner diameter, C18, 2 µm, 100 Å) fused to a silica nano-electrospray emitter (Dionex). The column was operated at a constant temperature of 35°C and the LC system coupled to a Q-Exactive mass spectrometer (Thermo Fisher Scientific). Chromatography was performed with a buffer system consisting of 0.1% formic acid (buffer A) and 80% acetonitrile in 0.1% formic acid (buffer B). The peptides were separated by a linear gradient of 3.8%–50% buffer B over 90 min at a flow rate of 300 nL/min. The Q-Exactive was operated in data-dependent mode with survey scans acquired at a resolution of 70,000 at m/z 200. Scan range was 300 to 2,000 m/z. Up to the top 10 most abundant isotope patterns with charge states + 2 to +5 from the survey scan were selected with an isolation window of 2.0 Th and fragmented by higher energy collisional dissociation with normalized collision energies of 30. The maximum ion injection times for the survey scan and the MS/MS scans were 250 ms and 50 ms, respectively, and the ion target value was set to 1E6 for survey scans and 1E5 for the MS/MS scans. MS/MS events were acquired at a resolution of 17,500. Repetitive sequencing of peptides was minimized through dynamic exclusion of the sequenced peptides for 20 s. The mass spectrometry proteomics data have been deposited to the ProteomeXchange Consortium via the PRIDE partner repository with the data set identifiers PXD041843 and 10.6019/PXD041843.

### Data processing and protein identification

All data were imported, aligned, and peak picked using Progenesis QI for Proteomics (Nonlinear Dynamics, version 4.1). Protein identifications were obtained by searching against Homo sapiens (Uniprot, UP000005640, December 2019) or RSV A2 (UP000181262, December 2019) UniProt databases using PEAKS studio 7 software (Bioinformatics Solutions Inc.) (56). Searches were conducted using a 1% false discovery rate, which was determined using a decoy database strategy. Peptide and fragment ion tolerances were set to 15 ppm and 0.02 Da, respectively. Two missed tryptic cleavages were permitted. Carbamidomethylation (cysteine) was set as a fixed modification. Oxidation (methionine) was set as a variable modification. Normalized abundance values based on the estimation of the amount of proteins present within samples were imported into MetaboAnalyst 4.0 for univariate and multivariate statistical analyses (57) All data were log transformed and unit scaled (whereby each variable was centered and divided by the standard deviation of the variable to convert to a z-score). PCA was used to reduce the high-dimensional data set and to explore initial data structure. Subsequently, different splits of the data (including comparisons based on time, on case-controls, and on cytosol versus membrane proteins) were analyzed in order to calculate *P*-values and fold changes. *P*-values were calculated by Student’s *t*-test or—in the case of three-way classifications—by analysis of variance. Protein signatures for each split of the data were constructed by including markers which met the criteria of having a false discovery rate (FDR)-adjusted *P*-value of <0.01. Proteins identified by only a single peptide were removed from analyses.

### Functional annotation and pathway analysis

Pathway analysis was carried out using a previously described method (58), using the lists of proteins characterized as significantly different between each cohort, where the threshold for significance was set at a *P*-value of <0.01. The analysis was performed using ClueGO (version 2.5.7), a plug-in application in Cytoscape (version 3.8.0) (59) searching against the Reactome pathway database. Over-representation analysis was used to compare the proportion of differentially expressed proteins within a pathway versus what would be expected by chance. This pathway over-representation analysis (including both enrichment and depletion) was done via a two-sided hypergeometric test and using Bonferroni step-down correction to calculate probabilities. Only pathways with FDR-adjusted *P*-value <0.01 and with a minimum of three proteins per pathway were considered. The major pathway identifiers (from the biomarker list) characterizing each cluster were highlighted in the generated visualizations.

### Immunoblotting analysis

Cells were lysed in SDS sample buffer (BIO-RAD) supplemented with β-mercaptoethanol (Sigma, Merck) and complete mini-EDTA-free protease inhibitors (Roche). Lysates were then boiled for 10 min, resolved by SDS-PAGE, and transferred to nitrocellulose membranes. Membranes were then blocked and probed for the indicated proteins. All primary antibody incubations were done overnight at 4°C.

### Fluorescent *in situ* hybridization

At 24 hpi, cells were incubated with medium supplemented with 20 µg/mL actinomycin D (Merck) for 1 h to inhibit cellular transcription. Cells were then fixed with 4% paraformaldehyde (PFA; Merck) in phosphate-buffered saline (PBS) for 15 min, permeabilized with 0.2% Triton X-100 in PBS for 5 min, before blocking with 1% bovine serum albumin (BSA) (Merck) in PBS supplemented with 4 µg/mL free streptavidin, for 1 h. Cells were then re-fixed with 4% PFA for 10 min and incubated at 37°C overnight in hybridization mix containing 2× saline-sodium citrate (SSC), 10% dextran, 1 mg/mL herring sperm DNA, 20% formamide (all purchased from Sigma, Merck), and 1 µM oligo dT probes, for the detection of total polyadenylated RNA (mRNA). The detection of viral NS1 and N mRNA was done using 10 µM total of a mix of probes (sequences in Table S1) in the hybridization mix as above, except with a formamide concentration of 50%. Cells were then washed at 42°C with 2× SSC plus 20% formamide for oligo dT probes and 50% formamide for all other probes. This was followed by washes in 2× SSC, then 1× SSC, and finally PBS. All probes were single-stranded DNA oligonucleotides synthesized with biotin at the 3´ end (Integrated DNA Technologies) and were detected using streptavidin-Alexa Fluor 488 conjugate (Life Technologies). Cells were then immunostained for viral proteins following the immunofluorescence staining protocol, before imaging by confocal microscopy.

### Confocal immunofluorescence microscopy

Cells were fixed and permeabilized as above before blocking with 1% BSA in PBS for 1 h. Primary antibody incubations were done overnight at 4°C followed by PBS wash and Alexa Fluor secondary antibody (Life Technologies) incubations, for 1 h at room temperature. Cells were then washed and mounted with Fluoromount mounting medium (Invitrogen, ThermoFisher Scientific) containing DAPI for nuclei staining. Fluorescence was imaged on a Leica Stellaris confocal microscope using 405 nm, 488 nm, and 568 nm laser lines for the appropriate dyes and a 63× oil immersion objective. Quantitation of fluorescence signal intensities along line regions of interest were exported from the LAS X software and graphs prepared in GraphPad Prism 9.

### Transmission electron microscopy

Infected cells growing on Thermanox coverslips (ThermoFisher Scientific, UK) were infected and fixed at the indicated times in phosphate-buffered 2% glutaraldehyde (Agar Scientific) for 1 h followed by 1 h in aqueous 1% osmium tetroxide (Agar Scientific). Cells were then dehydrated in an ethanol series: 70% for 30 min, 90% for 15 min, and 100% three times for 10 min. A transitional step of 10 min in propylene oxide (Agar Scientific) was undertaken before infiltration with a 50:50 mix of propylene oxide and epoxy resin (Agar Scientific) for 1 h. After a final infiltration of 100% epoxy resin for 1 h, the samples were embedded and polymerized overnight at 60°C. Next, 80-μm-thin sections were cut, collected onto copper grids (Agar Scientific), and grid stained using Leica EM AC20 before being imaged at 100 kV in a FEI Tecnai 12 TEM with a TVIPS F214 digital camera.

### Correlative light electron microscopy

Infected cells growing on gridded glass coverslips (MatTek) were fixed and labeled according to the described immunofluorescence method. Selected grid squares were imaged on a Leica TCS SP8 confocal microscope using 405 nm, 488 nm, and 568 nm laser lines for the appropriate dyes. The cells were then fixed in phosphate-buffered 2% glutaraldehyde (Agar Scientific) for 1 h, 1% osmium tetroxide (Agar Scientific) for 1 h, and 3% uranyl acetate (Agar Scientific) for 15 min before dehydrating in an ethanol series as described above. Next, cells were infiltrated with 100% epoxy resin (Agar Scientific) for 2 h, embedded and polymerized overnight at 60°C. Eighty micrometer-thin sections were cut from appropriate grid squares, collected unto copper grids (Agar Scientific), and grid stained using a Leica EM AC20 instrument. The specific cells imaged in the confocal were identified and imaged at 100 kV in a FEI Tecnai 12 TEM with a TVIPS F214 digital camera or a ThermoScientific Talos L120C G2 TEM with a Ceta 4K CMOS camera.

### Live imaging

Vero cells were seeded unto chambered number 1.5 borosilicate cover glass slide (Sigma, Merck) before transfection. Cells were transfected with plasmids expressing bRSV N-GFP and P in DMEM supplemented with 2% FCS. Sixteen-hour post transfection medium was replaced with Leibovitz’s L-15 medium without phenol red (Gibco, ThermoFisher Scientific), and imaged live with a Leica TCS SP8 confocal microscope using a 63× oil immersion objective. During imaging, cells were maintained at 37°C. Z-stacks were imaged for each time series and stacks acquired at intervals of 59 s.

### Image analysis and statistics

3D reconstruction was performed using Bitplane Imaris software v9.9.1 (Andor Technology PLC, Belfast, UK). The Surface creation tool was used to quantify the number of IBAGs and their volume, using the polyA signal as the source channel. Where required, touching objects were manually split and the “magic wand” option was used to add in objects that had not been properly segmented. The Surface tool was also used to quantify ribosome longest diameter using TEM images and IB volume and longest diameter, using either P or M2-1 signal as the source channel. As there is no direct Surface statistic for diameter within Imaris, the object-oriented (OO) bounding box statistic “BoundingBoxOO Length C” was used to give the length of the longest principal axis, thus the longest diameter of the object. Statistics were exported from Imaris and further processed in GraphPad Prism 9.

### Ribopuromycylation method

This method was performed according to the protocol described (60) with modifications. Cells infected with virus for 24 h were incubated with dimethyl sulfoxided (DMSO), 500 µM sodium arsenite (NaAsO_₂_), or 20 µM CPM for 1 h, before being left untreated or treated with 100 µg/mL CHX (Sigma, Merck) only for 5 min or 18.4 µM PRM (Sigma, Merck) and 100 µg/mL CHX for 5 min or 15 min at 37°C. Alternatively, infected cells were incubated with 18.4 µM PRM for 30 s, followed by incubation at 37°C with 100 µg/mL CHX and 18.4 µM PRM for 15 min. Cells were then fixed, permeabilized, and immunostained as described above or cell lysates were prepared using Laemmli sample buffer for SDS-PAGE analysis. Densitometry analysis was performed using the ImageLab software.

### Immunoprecipitation

Cells transiently expressing M2-1-GFP or GFP as control were lysed with cell lysis buffer (CST) supplemented with complete mini-EDTA-free protease inhibitors (Roche) on ice and cell debris removed by centrifugation. Lysates were then incubated at 4°C overnight with GFP-Trap agarose (Chromotek) or Dynabeads (Invitrogen, ThermoFisher Scientific) that were pre-incubated for 30 min at room temperature with anti-eIF4G antibody, following the manufacturer’s protocols. After being washed, samples were eluted in Laemmli buffer supplemented with β-mercaptoethanol, boiled for 10 min, and resolved by SDS-PAGE.

## Data Availability

The data to support the findings of this study are included within the article and it’s supplemental figures and data sets. The proteomics data are available via ProteomeXchange with identifier PXD041843.

## SUPPLEMENTAL MATERIAL

Supplemental Data Set jvi.00153-24-s0001.xlsx Ion abundance data, *P* value and fold change analysis for cytosol fraction, reactome pathway analysis results, and sorted data.

Supplemental material jvi.00153-24-s0002.docx Figures S1 to S7, Table S1, and legends for Movies S1 and S2.

Movie S1 jvi.00153-24-s0003.mp4 Imaris reconstruction of confocal image Z-stacks of an RSV-infected cell.

Movie S2 jvi.00153-24-s0004.mp4 Imaris reconstruction of confocal image Z-stacks of an RSV-infected cell.

ASM does not own the copyrights to Supplemental Material that may be linked to, or accessed through, an article. The authors have granted ASM a non-exclusive, world-wide license to publish the Supplemental Material files. Please contact the corresponding author directly for reuse.
