# Supplementary material for: Viral PIC-pocketing: RSV sequestration of translational preinitiation complexes into bi-phasic biomolecular condensates
Source: J Virol. 2024 Feb 29;98(3):e00153-24. doi: 10.1128/jvi.00153-24 (PMC10949503; doi:10.1128/jvi.00153-24)

**Supplementary Figures:**


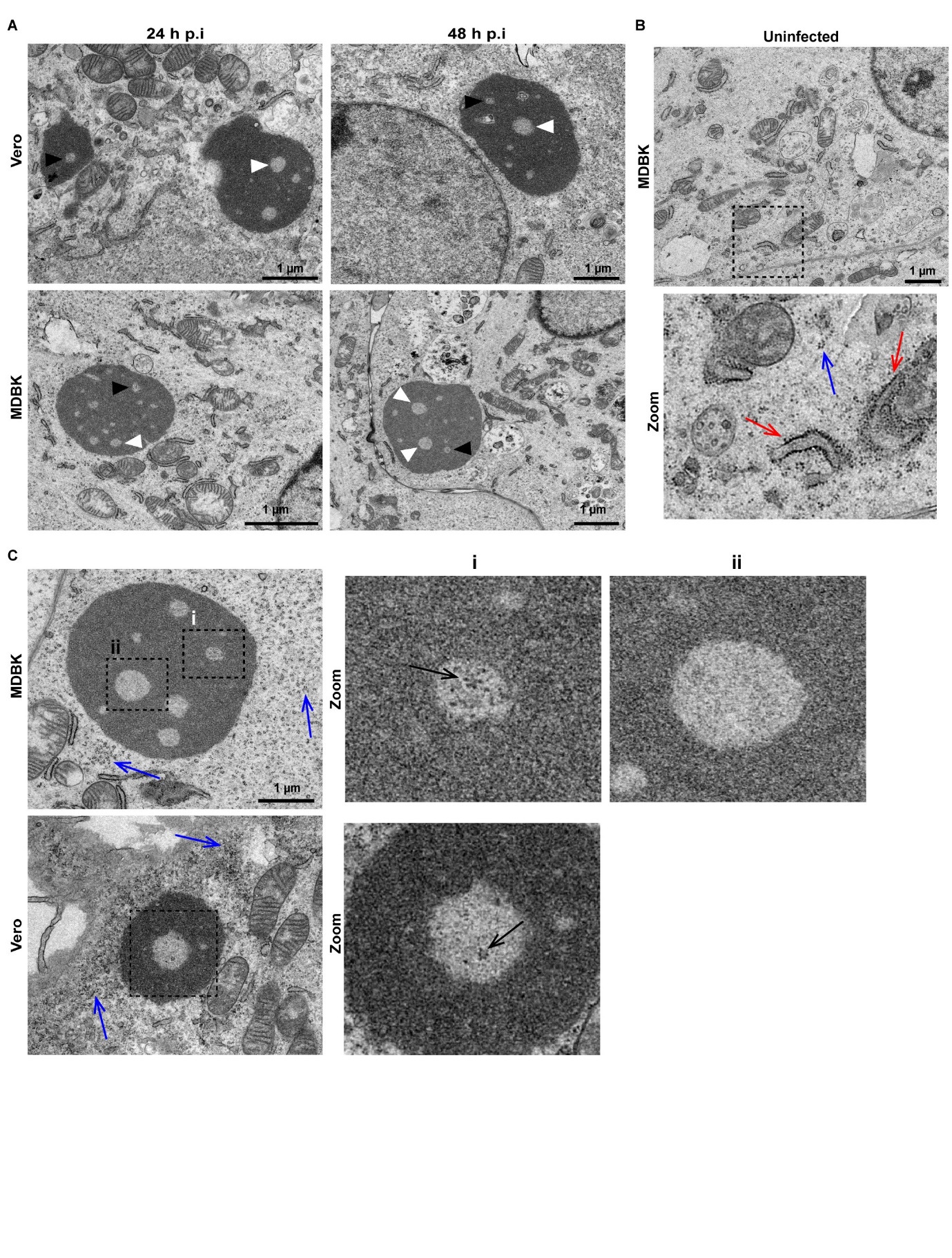


**Supplementary figure 1.** (A) Vero and MDBK cells were infected with bRSV for 24 and 48 h, fixed in glutaraldehyde and processed for TEM as detailed in the methods. Representative images are shown from the indicated time points. Black arrow heads indicate microdomains with concentration of ribosomes and white arrow heads to those with minimal ribosomal content. (B) TEM of uninfected MDBK cell. Red arrows within zoom of the boxed area indicate ribosomes on the RER and blue arrow, ribosomes in the cytoplasm. (C) Multiphasic IBs from bRSV infected MDBK and Vero cells showing more examples of the heterogeneity in Ribosome presence.

**
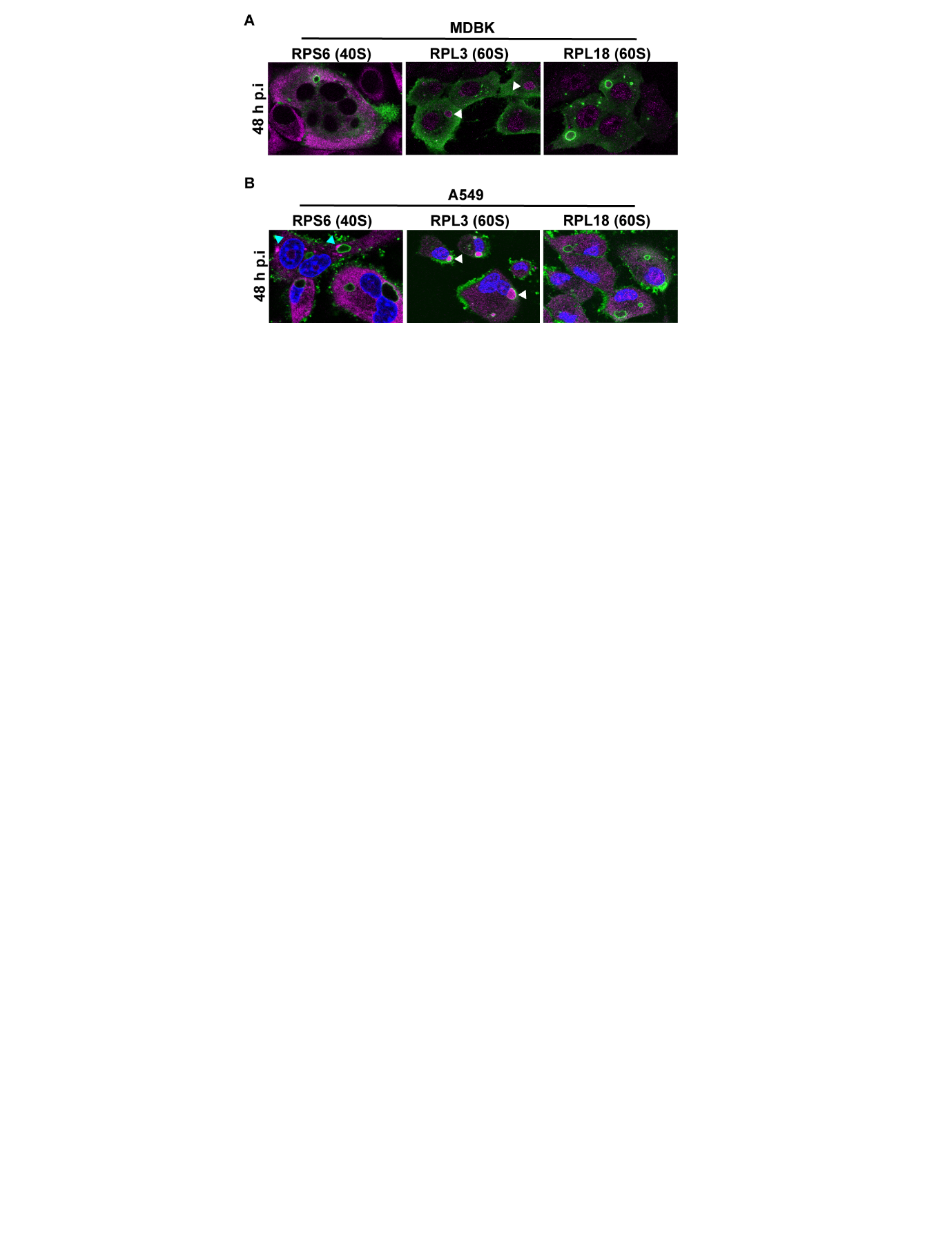
Supplementary figure 2.** (A and B) Uninfected or cells infected with RSV (A; bRSV-infected MDBK cells and B; hRSV-infected A549 cells) at an MOI of 2 for 48 h were fixed, permeabilised and immuno-stained with antibodies against RSV N or P protein (green), ribosomal subunits, RPS6, RPL3 and RPL18 (magenta), and nuclei stained with DAPI (blue).

**
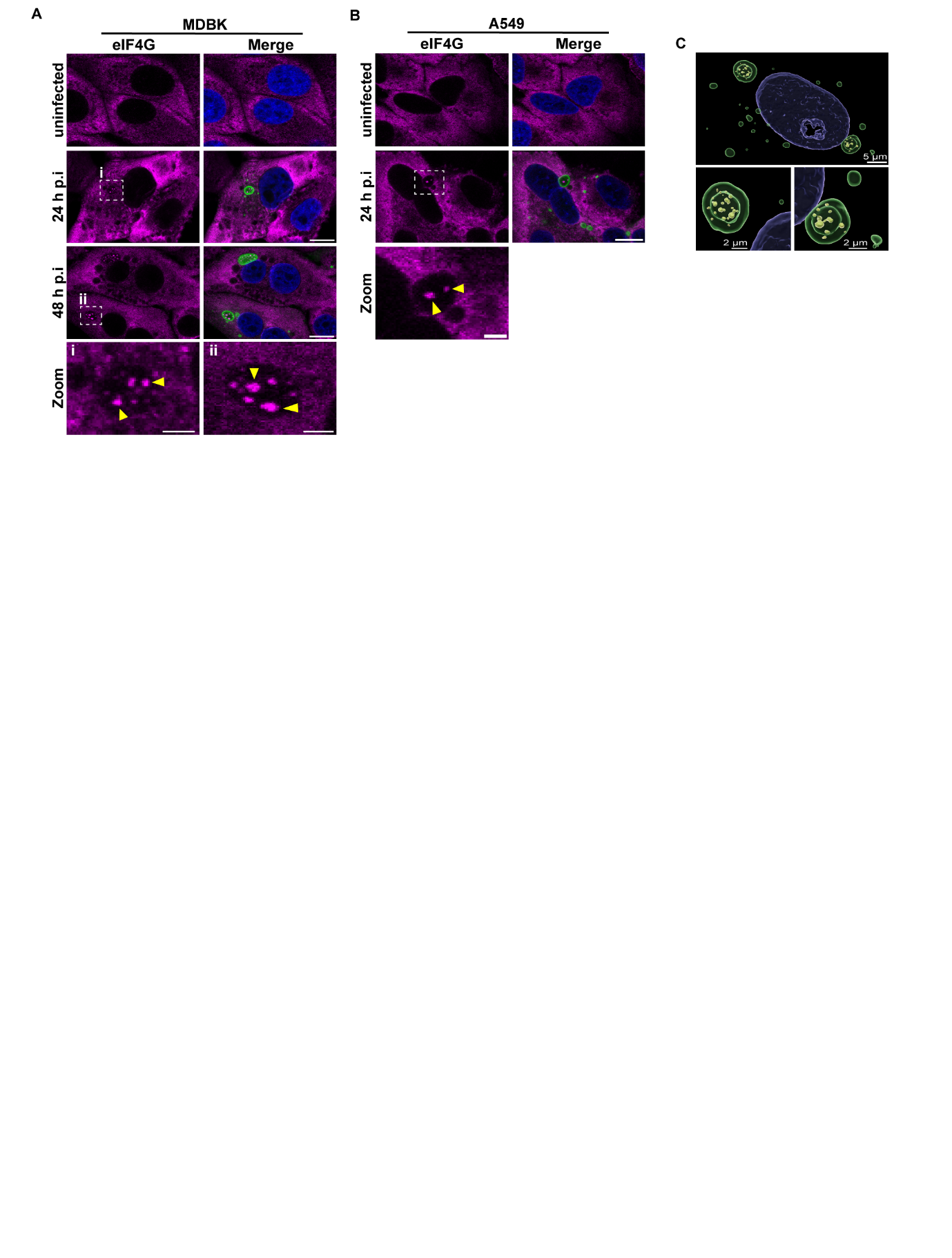
**

**Supplementary figure 3.** (A) MDBK and (B) A549 cells respectively infected with bRSV or hRSV at an MOI of 1 for the indicated times, were fixed and immuno-stained for eIF4G (magenta) and RSV N (green) followed by confocal imaging. Zoom panels are enlarged images of the boxed areas; labelled i and ii in A and unlabelled in B. Scale bars, 10 µm in main and 2 µm in zoom. (C) Imaris reconstruction of confocal image Z-stacks of bRSV infected Vero cells FISH stained for total PolyA mRNA (yellow) and immuno-stained for RSV P (green). Lower panels are enlarged images of the IBs from the top panel.

**
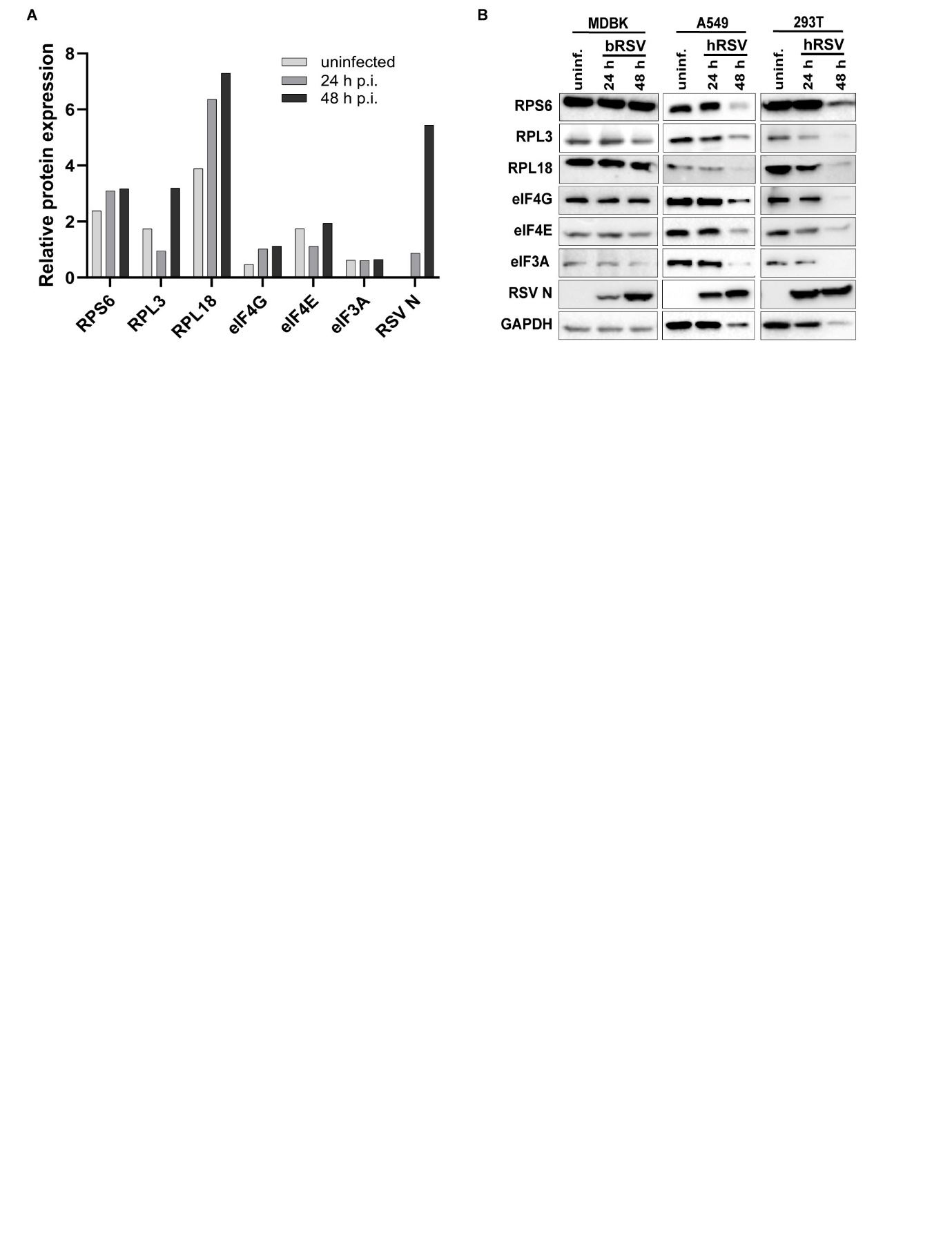
**

**Supplementary figure 4.** (A) Densitometry analysis of the immunoblots shown in figure 4B. Protein expression levels are expressed relative to GAPDH loading control. (B) Immunoblots showing levels of the indicated proteins in whole cell lysates prepared from uninfected (uninf.) or from bRSV- or hRSV-infected (24 or 48 h p.i.) MDBK, A549 or 293T cells, respectively.

**
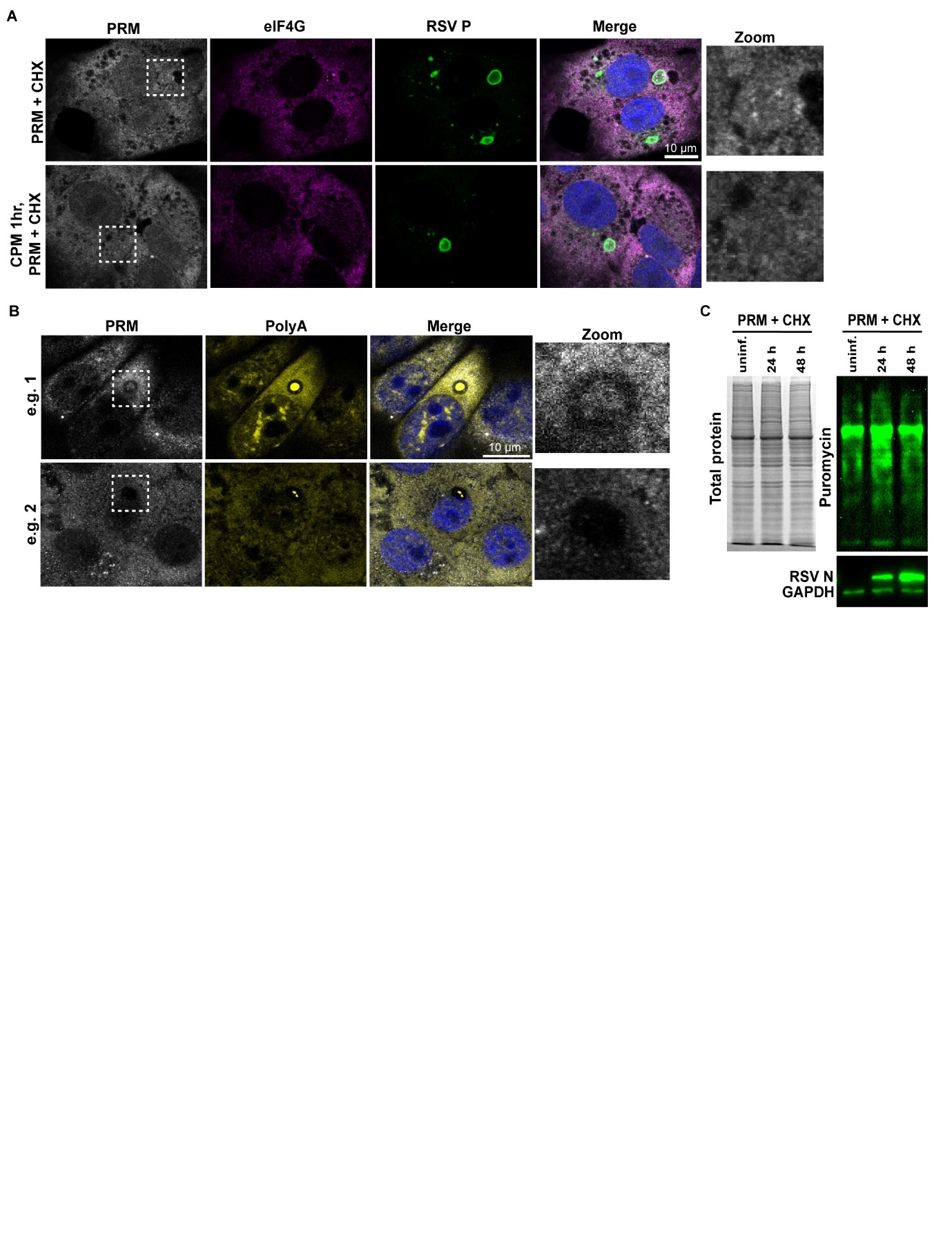
Supplementary figure 5.** (A) bRSV-infected Vero cells grown on coverslips were treated with DMSO or cyclopamine (CPM) for 1 hr, followed by CHX and PRM for 5 mins. Cells were fixed and immuno-stained with anti-PRM, eIF4G and RSV P antibodies. (B) Vero cells infected with bRSV for 24 h were pulsed with PRM for 30 secs before the addition of CHX and then incubated for 15 mins at 37°C. FISH staining was then performed to detect PolyA mRNA, followed by immunostaining for PRM or only immuno-stained for PRM and eIF4G. (C) Vero cells left uninfected or infected with bRSV for 24 or 48 h were treated with PRM for 30 secs followed by CHX for 15 mins. Whole cell lysates were then prepared for Coomassie staining to assess total protein levels or levels of ribopuromycylated products by immunoblotting with anti-PRM mAb. RSV N was detected to confirm infection and GAPDH as an internal loading control. Three independent repeats of the presented data were performed.

**
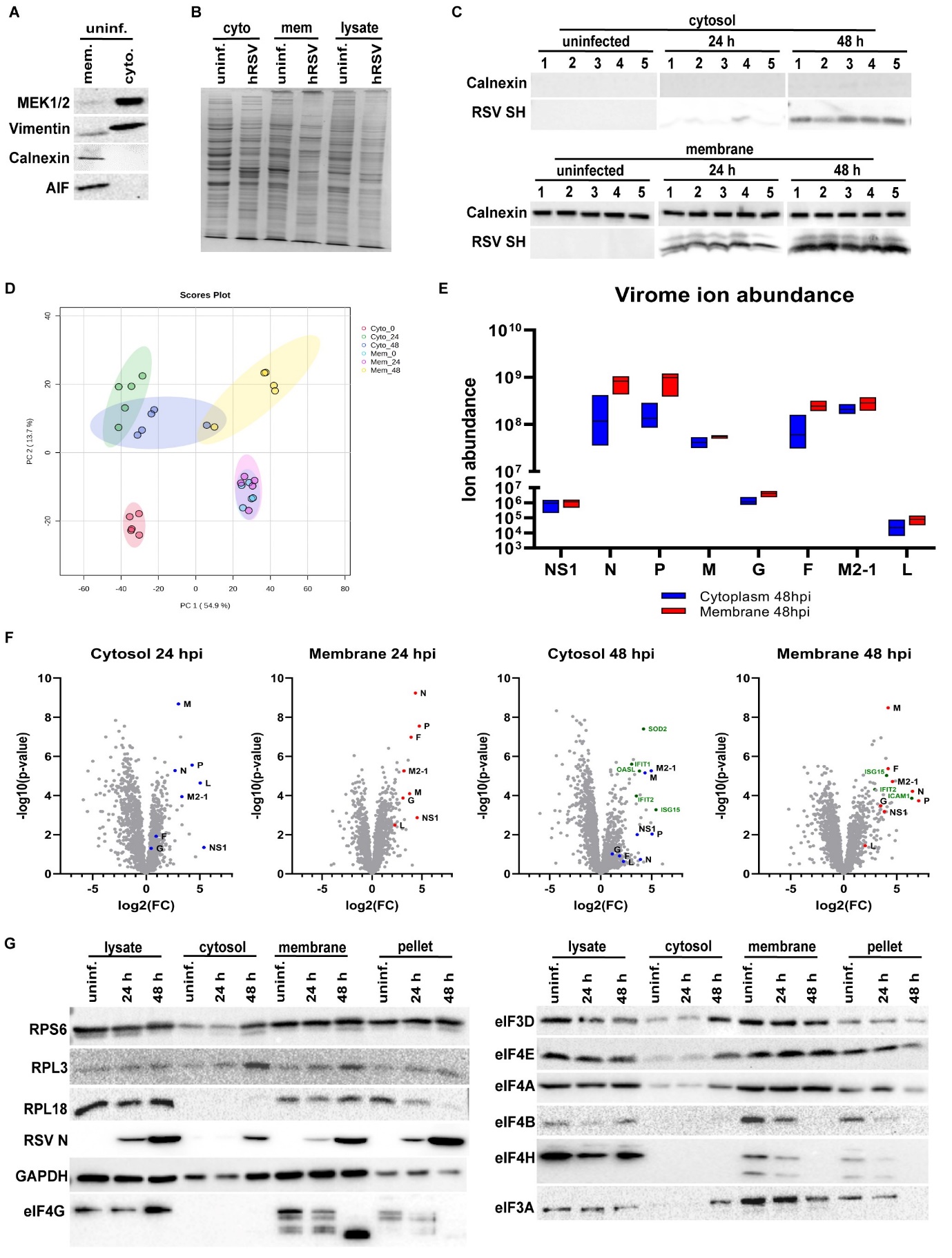
**

**Supplementary figure 6.** (A) Validation of fractionation kit. Cytosolic and membrane fractions of uninfected A549 cells were separated using a Thermofisher Mem-PER™ Plus membrane protein extraction kit. Immunoblotting was performed on the separated fractions to detect marker proteins of known cellular location. (B) Sub-cellular fractions were made of uninfected or hRSV-infected A549 cells and fractions analysed by SDS-PAGE and coomassie staining. (C) Quintuplicate samples (cytosol and membrane) of fractions analysed by mass spectrometry were immunoblotted for the indicated protein markers. (D) Normalised and log transformed ion abundancy data from the 30 fraction samples were analysed by principal component analysis (PCA) to show the distribution of the samples analysed when the feature space is reduced to two dimensions. The PCA axes show the first and second most important components in terms of the reduced space along which the samples show the largest variation. The units of the axes are percent variation in the overall dataset explained by the two components. (E) Ion abundancies for positively detected hRSV proteins are shown in the respective membrane (Mem) and cytoplasmic (Cyto) fractions at 48 hpi. Each floating boxplot represents the minimum and maximum abundancies observed from the 5 biological replicates, with the line representing the mean. NS2, M2-2 and SH were not detected. (F) Volcano plots reflecting comparison of ion abundancies between mock and infected membrane and cytosolic fractions at 24 and 48 h p.i. Fold changes (log2) and p-values (-log10 transformed) are plotted. The eight successfully detected hRSV proteins are separately highlighted in blue (cytosol-associated) or red (membrane) together with some exemplar host proteins, e.g. IFIT1. All other proteins are represented by grey symbols. (G) Immunoblots showing levels of the indicated proteins in whole cell lysates (lysate), as well as in cytosolic (cytosol) or membrane fractions and the remaining pellet following fractionation, prepared from uninfected (uninf.) or from hRSV-infected (24 or 48 h p.i.) A549 cells.

**
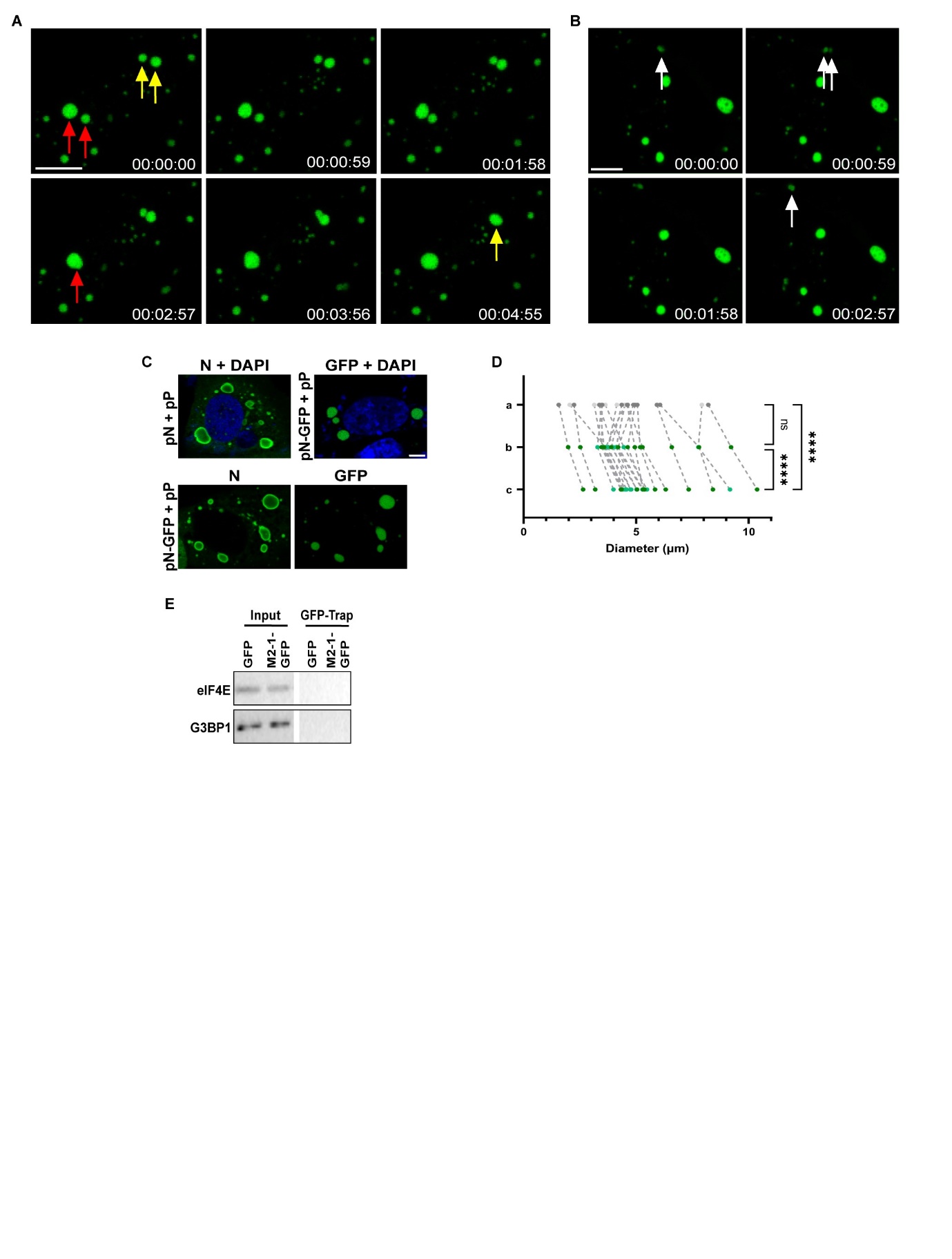
**

**Supplementary figure 7.** (A and B) IB fusion and fission. Time-lapse imaging of pseudo-IBs (green) formed in Vero cells transfected with plasmids expressing RSV N-GFP and P. Cells were imaged at around 16 h post tran sfection at 59 sec intervals in a chamber maintained at 37°C, using a Leica Stellaris confocal microscope. Red and yellow arrows in A indicate pseudo-IBs that eventually fuse together. White arrows in B indicates an IB undergoing fission and subsequent fusion. Images are representative of three independent experiments. Scale bars, 10 µm. (C) Pseudo-IBs formed in Vero cells transfected with plasmids expressing RSV N and P or N-GFP and P. Detection of RSV N protein with an anti-N mAb shows the characteristic ring pattern whereas, GFP is even distributed through-out the pseudo-IB. Scale bar, 5 µm. (D) Comparison of bRSV IB diameters in Vero cells measured from EM and IF images as described in Fig 6D. One-way ANOVA with Tukey’s multiple comparison was used for statistical analysis; *****P*<0.0001, ns; non-significant. (E) Immunoblots showing levels of eIF4E and G3BP1 in whole cell lysates (input) and pull-downs following GFP-TRAP.

**Supplementary information video 1**

Imaris reconstruction of confocal image Z-stacks of an RSV infected cell pseudo-coloured as follows: DAPI (purple), anti-M2-1 antibody (light blue) and an olidT FISH probe (yellow). 3D reconstruction was performed using Bitplane Imaris software v9.9.1 (Andor Technology PLC, Belfast, UK) – see materials and methods for more detail.

**Supplementary information video 2**

Imaris reconstruction of confocal image Z-stacks of an RSV infected cell pseudo-coloured as follows: DAPI (purple), anti-P antibody (green) and an olidT FISH probe (yellow). 3D reconstruction was performed using Bitplane Imaris software v9.9.1 (Andor Technology PLC, Belfast, UK) – see materials and methods for more detail.


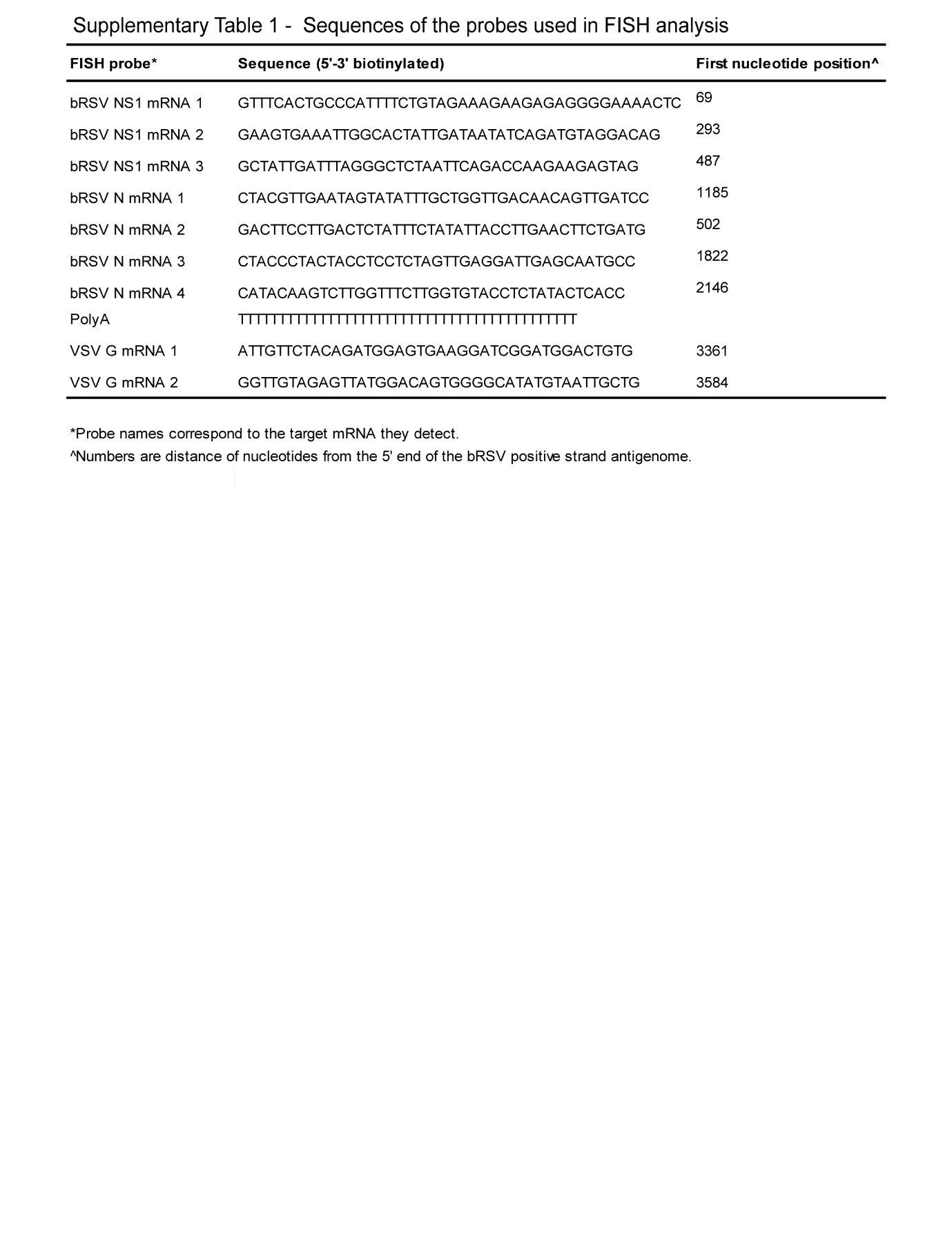

Supplement: Supplemental material — Figures S1 to S7, Table S1, and legends for Movies S1 and S2. [file jvi.00153-24-s0002.docx]
